# Machine learning-based risk factor analysis and prevalence prediction of intestinal parasitic infections using epidemiological survey data

**DOI:** 10.1371/journal.pntd.0010517

**Published:** 2022-06-14

**Authors:** Aziz Zafar, Ziad Attia, Mehret Tesfaye, Sosina Walelign, Moges Wordofa, Dessie Abera, Kassu Desta, Aster Tsegaye, Ahmet Ay, Bineyam Taye

**Affiliations:** 1 Colgate University, Department of Mathematics, Hamilton, New York, United States of America; 2 Colgate University, Department of Biology, Hamilton, New York, United States of America; 3 Colgate University, Department of Computer Science, Hamilton, New York, United States of America; 4 Addis Ababa University, College of Health Sciences, Department of Medical Laboratory Science, Addis Ababa, Ethiopia; Institute of Tropical Medicine, BELGIUM

## Abstract

**Background:**

Previous epidemiological studies have examined the prevalence and risk factors for a variety of parasitic illnesses, including protozoan and soil-transmitted helminth (STH, e.g., hookworms and roundworms) infections. Despite advancements in machine learning for data analysis, the majority of these studies use traditional logistic regression to identify significant risk factors.

**Methods:**

In this study, we used data from a survey of 54 risk factors for intestinal parasitosis in 954 Ethiopian school children. We investigated whether machine learning approaches can supplement traditional logistic regression in identifying intestinal parasite infection risk factors. We used feature selection methods such as InfoGain (IG), ReliefF (ReF), Joint Mutual Information (JMI), and Minimum Redundancy Maximum Relevance (MRMR). Additionally, we predicted children’s parasitic infection status using classifiers such as Logistic Regression (LR), Support Vector Machines (SVM), Random Forests (RF) and XGBoost (XGB), and compared their accuracy and area under the receiver operating characteristic curve (AUROC) scores. For optimal model training, we performed tenfold cross-validation and tuned the classifier hyperparameters. We balanced our dataset using the Synthetic Minority Oversampling (SMOTE) method. Additionally, we used association rule learning to establish a link between risk factors and parasitic infections.

**Key findings:**

Our study demonstrated that machine learning could be used in conjunction with logistic regression. Using machine learning, we developed models that accurately predicted four parasitic infections: any parasitic infection at 79.9% accuracy, helminth infection at 84.9%, any STH infection at 95.9%, and protozoan infection at 94.2%. The Random Forests (RF) and Support Vector Machines (SVM) classifiers achieved the highest accuracy when top 20 risk factors were considered using Joint Mutual Information (JMI) or all features were used. The best predictors of infection were socioeconomic, demographic, and hematological characteristics.

**Conclusions:**

We demonstrated that feature selection and association rule learning are useful strategies for detecting risk factors for parasite infection. Additionally, we showed that advanced classifiers might be utilized to predict children’s parasitic infection status. When combined with standard logistic regression models, machine learning techniques can identify novel risk factors and predict infection risk.

## Author summary

In developing countries such as Ethiopia, intestinal parasites are a significant public health problem. These parasites are detrimental to the health of schoolchildren. Numerous risk factors for parasitic infections have been identified using uni- and multi-variate logistic regression. However, logistic regression has inherent limitations when applied to data sets with a large number of risk factors. We used machine learning techniques in conjunction with logistic regression models to identify relevant risk factors for parasitic infections in a dataset of 954 Ethiopian schoolchildren with 54 different risk factors for parasitic infections. Additionally, we developed predictive models of parasitic infection. Compared to logistic regression, we discovered that machine learning techniques identified novel risk factors and had higher predictive accuracy. Furthermore, we discovered that infection prediction could be aided by combining socioeconomic, health, and hematological characteristics. As a result, we concluded that advanced machine learning methods should be used in conjunction with logistic regression to study parasitic infections.

## Introduction

Gastrointestinal parasitic infections, caused by protozoans and helminths, are endemic to developing countries [1]. The prevalence of such parasites is highest in children in sub-Saharan Africa [2]. For instance, in Ethiopia, intestinal parasitic infections continue to be a widespread public health problem, with a prevalence of around 25% for protozoal infections and 21.7% for soil-transmitted helminths [3]. Intestinal protozoans include *Giardia lamblia* and *Entamoeba histoytica*, and soil-transmitted helminths include roundworms (*Ascaris lumbricoides* and *Strongyloides stercoralis*), hookworms (*Ancylostoma duodenale* and *Necator americanus*), and the human whipworm (*Trichuris trichiura*). These parasites can cause serious health problems such as lack of appetite, anemia, decreased physical growth, and impaired intellectual development [4–7].

Previous literature suggests that risk factors for intestinal parasites include a combination of socioeconomic, demographic, and environmental factors [8, 9]. These studies often use standard statistical tests like logistic regression and frequency analysis [10]. These approaches, however, may have drawbacks such as overfitting the data when applied to data sets with a large number of highly correlated variables. As a result, they may not accurately depict the relationship between risk factors and disease outcomes [11].

Advanced machine learning approaches are based on the premise that computers can mine complex patterns and interactions from data sets based on mathematical rules and statistical assumptions [12]. In contrast to an epidemiological or statistical approach, machine learning does not rely on strong assumptions about the data’s linearity or predictor variables’ mutual dependence but rather on iterative computing techniques to learn from massive data sets. Recent studies have used various machine learning approaches to accurately predict and identify relevant risk factors for disease outcomes like heart failure, acute renal failure, Type 2 diabetes, and malaria [13–16]. However, effective algorithms, such as association rule learning, have not been widely used in disease risk factor analysis. Historically, association rule learning has been used in market basket analysis to determine consumer behavior in supermarkets [17]. Although this strategy may have novel applications in the field of epidemiology, it has been used in only a few studies to date; when applied to public health data sets, association rule learning was capable of identifying patterns of disease co-occurrences [18].

To our knowledge, no study has attempted to combine multiple machine learning techniques to investigate risk factors and make accurate predictions for intestinal parasitic diseases. Thus, our analysis could provide important information for directing targeted public health interventions for intestinal parasitosis prevention and awareness. In this study, we used data from a comprehensive Ethiopian school survey to identify risk factors for parasite infections and to create predictive prevalence models utilizing powerful machine learning techniques. Additionally, we employed association rule learning to find combinations of risk factors that contribute to the development of a particular infection. Our findings demonstrate the potential of machine learning in epidemiology.

## Methods

### Ethics statement

We obtained written or fingerprint consent from children’s parents or their legal guardians after informing them of the study procedures. To ensure participant privacy, confidential numerical identifiers were assigned to each child and all participant information remains password protected in electronic files. The children were also informed about their ability to withdraw from this study at any time without jeopardizing their right to receive any services at their school. Children who were found to have intestinal parasites were treated with anti-parasitic drugs in local health centers. Departmental Research and Ethics Review Committee (DRERC) of Addis Ababa University College of Health Sciences, Department of Medical Laboratory Sciences, approved the study.

#### Data collection

In 2016 and 2017, we conducted surveys among school children in Ethiopia’s Oromia region. We surveyed five elementary schools: Abdi Boru, Laga Dima, and Wasarbi in the town of Sululta, and Batu and Sher in the town of Ziway. We obtained written consent from the parents or legal guardians of the children and used an interviewer-led questionnaire. We collected data from a total of 954 schoolchildren. Additionally, we tested each child’s stool and blood for parasite infection. Within 30 minutes of collection, fecal samples were analyzed using direct wet mount microscopy and the Kato-Katz technique. Any remaining fecal samples were analyzed using formol-ether concentration analysis at the Department of Medical Laboratory at Addis Ababa University, Ethiopia. A more in-depth description of each parasitological test can be found in our previously published study [19]. Allergic disorders related questions were derived from the widely used and validated ISAAC symptoms questionnaire [20], which had previously been used in this age group of children [21, 22] in Ethiopia.

#### Risk factors surveyed

We collected data on demographic, socioeconomic, health related, environmental, and hematological variables. Table 1 contains the comprehensive list of risk factors.

**Table 1:** Risk factors for intestinal parasitic infections grouped by categories such as demographic, socioeconomic, health, environmental, and hematological factors.

Demographic Factors	Socioeconomic Factors	Health Factors	Environmental Factors	Hematological Factors
Age	Sleeps on a bed	Cockroach skin	Application of dung to farm fields	Hematocrit
Deworming	Household burns	Prick test	Cigarette smokers in the house	Hemoglobin
Family Size	Charcoal	Child has asthma	Location of cooking area	Lymphocytes count
Residence	Household burns	Child has hay	Family has a cat	Mean Corpuscular Hemoglobin
Sex	Dung	Fever	Family has a cow	Mean Corpuscular Hemoglobin Concentration
	Household burns gas	Child has had hay	Family has a dog	
	Household burns leaves	fever in last year	Family has a hen	Mean Corpuscular Volume
	Household burns nafta	Child with rash in last year	Family has a horse	Platelet count
	Household burns wood	Child has wheeze in last year	Family has a pig	Red Blood Cell count
	Household uses electricity	Father with asthma	Family has a sheep	White Blood Cell count
	Composition of floor in the home	Father with hay fever	Source of water in household	
	Maternal Education	Father with wheeze	Type of toilet in the home	
	Maternal Occupation	Mother with asthma	Location of household waste disposal	
	Child’s mattress	Mother with hay fever		
	Roof on the home	Mother with wheeze		
	Composition of walls in the home			
	What the child sleeps on			

#### Outcome definition

For each sample, we classified infections as binary (infected or not infected) for each of four outcomes: (1) any STH (with "infected" testing positive for any *A*. *lumbricoides*, *T*. *trichiura*, *A*. *duodenale*, *N. americanus*, or *S*. *stercoralis*), (2) helminths (with "infected" testing positive for any eggs or larvae of any helminthic parasites), (3) protozoans (*G*. *lamblia* or *E*. *histolytica*), or (4) any parasite infection (positive for any protozoan and helminthic parasites).

#### Data processing

Any sample that lacked a value for one of the outcome variables (n = 12) in our investigation was omitted from the data set. We also excluded risk factors with a missing value rate greater than 5% across all samples surveyed. Additionally, we used one-hot encoding to convert risk factors with more than two categories to multiple factors and eliminated one category for each factor to avoid multicollinearity in our data set. Following data processing, we had 942 samples with 68 risk factors for each infection outcome. Finally, we performed feature scaling (a.k.a. min-max normalization) on continuous variables to standardize them to a range of 0 to 1 for data imputation and data balancing (discussed later).

#### Logistic regression and statistics

We performed univariate and multivariate logistic regression for each infection outcome. We compared risk factors identified through univariate and multivariate logistic regression to those identified using four machine learning feature selection methods. Due to the fact that logistic regression models require multiple hypothesis testing, we utilized the Benjamini-Hochberg p-value correction, which limits the rate of false discovery to 5% [23].

#### Feature selection

We employed feature selection algorithms to identify and eliminate redundant risk factors for each infection outcome (dependent variable). We used the ranking-based approaches Information Gain (IG) and ReliefF (ReF) to determine the importance of each risk factor (also referred to as feature throughout the paper) independently from the other risk factors in the data set. IG calculates information gain for each risk factor for the infection outcome. The risk factors contributing the most information for the infection outcome have a higher information gain and are selected [24]. ReF determines the risk factor’s significance by randomly selecting samples and computing the Manhattan distance between neighboring samples and their disease outcomes [25]. We also used subset-based approaches such as Joint Mutual Information (JMI) and Minimum Redundancy Maximum Relevance (MRMR) to identify relevant risk factors. These subset-based methods identify risk factors with a higher mutual information score with the outcome than the mutual information between the selected risk factors [26, 27]. To avoid bias in our classification runs, we performed feature selection on the training data and filtered the validation/test data using the same features. We then ranked our risk factors using ranking or subset-based scoring metrics and included the top twenty features in our classifier. The strength of risk factors for each outcome was determined by their occurrences across multiple feature selection methods as well as univariate and multivariate logistic regression. We defined consensus as a feature being in the top twenty for at least 95% feature selection runs.

#### Classifiers and hyperparameters

To ensure the robustness of our findings, we used a variety of classifiers, machine learning methods that model the relationship between risk factors and infection outcomes. We used tree-based methods like Random Forests (RF) and XGBoost (XGB) and linear models like Support Vector Machines (SVM) and Logistic Regression (LR) [28–30]. We evaluated the performance of our classifiers using accuracy scores and area under receiver operating characteristic (AUROC) curves. Classifiers incorporate a variety of hyperparameters that must be customized for each dataset. As a result, we used preliminary testing to determine an appropriate range of hyperparameters for each classifier, followed by grid searching to determine the optimal combination of hyperparameters for maximizing accuracy. For Logistic Regression classifier, we used no penalization method and utilized the default optimizer, Large-scale Bound-constrained Optimization (L-BFGS-B) [31].

#### Data imputation

Data collection through questionnaires or surveys frequently results in missing or inconsistent data [32]. Missing values hinder our ability to conduct unbiased data analysis and negatively affect machine learning algorithms, including feature selection and classification [33]. Simply eliminating rows of data with a single missing value can result in skewed statistical results and a decrease in statistical power. We used k-nearest neighbors’ imputation to overcome this issue, which finds missing values and estimates them using a number, k, of nearest neighbors belonging to the same class [34]. We chose a value of 5 for k based on empirical evidence. KNN imputation has been shown to reduce the bias for feature selection approaches such as ReliefF (ReF) [35]. Imputation was done only on training data samples.

#### Model validation

We employed stratified tenfold cross-validation to determine each model’s generalizability. We divided the data set into ten folds (subsets) for each combination of feature selection method and classifier, maintaining a consistent distribution of our outcome class for each fold. Then, we performed the k-nearest neighbors’ imputation for each fold. To ensure robust results, we did the cross-validation ten times using a different random number generator seed each time.

#### Class imbalance

If a dataset is unbalanced, the feature selection and classification models tend to overfit to the majority outcome. As a result, the model may be inaccurate. We employed Synthetic Minority Oversampling (SMOTE) technique to increase model accuracy by balancing the unbalanced dataset. SMOTE accomplishes this by identifying the k-nearest neighbors (we used k = 5 based on empirical evidence) and randomly generating new data along the line between two neighbors of the same class [36].

SMOTE is increasingly being employed in epidemiological research with highly skewed data sets. For example, SMOTE improved the prediction accuracy of decision tree models on a data set of patients with chronic kidney disease [37]. Additionally, SMOTE was utilized in conjunction with a Random Forest classification technique to distinguish between patients and non-patients in a cervical cancer data set to increase the overall accuracy after balancing [38]. In our investigation, to avoid bias, we used SMOTE to oversample the number of cases for each of the four outcomes in order to balance the training data set. We ran our classification runs with and without SMOTE to determine whether balancing improved our models. We also used under sampling on our training sets. However, due to the small number of outcome variables, we obtained lower accuracies than without any data balancing or with SMOTE.

#### Association rule learning

We used association rule learning to deduce risk factor combinations that had a strong connection with the four disease outcomes [39]. The technique employs a metric called "support" that quantifies a rule’s frequency of occurrence as a proportion of all samples, as well as "confidence," a metric that quantifies the likelihood that the antecedent (e.g., a subset of risk factors) caused the consequent (e.g., outcome). As a proxy for the association rule’s strength, we use lift, the ratio of observed to expected support when the antecedent and consequent are unrelated. A lift greater than one indicates that the antecedent is likely to result in the consequent. We chose a lift of two as our cutoff value and used rules with the highest support and confidence values. In the event of tied lift values, we identified rules that contained risk factors in greater than 20% of the top association rules.

#### Code availability

The study’s code was written in Python and R due to their user-friendliness and advanced statistical learning libraries. Our code can be found at https://github.com/Ziad-Attia/Machine-Learning-Package.git.

## Results

### Characteristics of the study population

In our study of 942 children, 54.8% (516) were female, 55.7% (525) were urban residents, 9.2% (87) were younger than 6, 43.4% (408) were between the ages of 6 and 10, and the remaining were older than 10. 79.5% (749) of children were dewormed. We found that 5.1% of children were infected with STH (48 cases), 5.8% (55) with protozoans, 15.1% (142) with any helminth, and 20.1% (189) with any parasite.

### Logistic regression

Multivariate logistic regression models showed a significant decrease in the odds of having an STH infection among children who lived in cities versus villages and were dewormed (p < 0.05). On the other hand, we found a significant increase in the odds of having STH infection for children belonging to households that sometimes used nafta for cooking, and in children whose fathers had hay fever (p < 0.05). (S1 Table contains all odds ratios and p-values).

A separate logistic regression using any protozoan infection as the outcome was significantly related to paternal wheezing, sometimes burning leaves, and having a dog (p < 0.05). In addition, cooking in the home and child’s wheezing was found to significantly decrease the odds of having a protozoal disease (p < 0.05). (S2 Table contains all odds ratios and p-values).

Furthermore, any helminth infection was significantly associated with paternal hay fever, maternal occupation, family size, mean corpuscular hemoglobin concentration, hematocrit levels, and type of mattress on which the child sleeps (p<0.05). (S3 Table contains all odds ratios and p-values for this infection).

Lastly, looking across any parasite (defined positive either protozoa or helminths) infections, we found few demographic and lifestyle factors such as family size, increase in hematocrit were inversely associated with any parasite infection, while a significant increase in odds of any parasite infection was found with disposing of waste in an open field, paternal hay fever, and a positive dust mite skin prick test. (S4 Table contains all odds ratios and p-values for this infection). However, after Benjamini-Hochberg correction, all of the aforementioned risk factors lost statistical significance due to the high dimensionality of the data. This demonstrates the value of combining advanced machine learning-based feature selection methods with traditional logistic regression once more.

### Risk factor analysis

Feature selection methods provided a novel and complementary approach to logistic regression for risk factor analysis. Table 2 compares the risk factors identified by feature selection approaches to the significant risk factors identified through logistic regression models. For each respective feature selection method, a risk factor was considered important if it appeared in at least 95% of the runs performed. We found nafta burning (frequent and infrequent), frequent burning of leaves, having a positive cockroach skin prick test, and father with wheeze as strong predictors for all outcome variables. These risk factors were present in at least eight feature selection or logistic regression models. Risk factors such as, child with asthma in last year, source of water, and household with thatched roof, mother with hay fever, platelets’ count, and having a pig were identified across all infection outcomes using eight feature selection methods but were not identified using logistic regression models. (Table 2). We observed some variations in selected risk factors for each outcome variable. For example, frequent dung burning was identified by feature selection for STH infections, but not or any parasitic infections. Cooking inside living area and some leaves burning were identified by feature selection and logistic regression for predicting protozoans, but not for any STH infections (Table 2). Furthermore, our feature selection model frequently identified none statistically significant features in logistic regressions. In contrast, logistic regressions identified only two features of STH infection that were missed by feature selection.

**Table 2:** Risk factors for each infection outcome based on univariate (U) and multivariate (M) logistic regression (α = 0.05) and the feature selection methods: InfoGain (IG), ReliefF (ReF), Joint Mutual Information, and Minimum Redundancy Maximum Relevance (MRMR). Robustly selected features (appeared in 95% of the top 20 of 100 feature selection runs for each method). Risk factors are ranked according to their frequency of occurrence in three approaches. Upwards arrows indicates significant odds ratio greater than 1, and downwards arrows indicate odds ratio lesser than 1. Arrows with a * lost statistical significance after Benjimini-Hochberg p-value adjustment.

	STH Infection						Protozoan Infection						Parasitic Infection						Helminthic Infection					
Risk Factor	IG	ReF	MRMR	JMI	U	M	IG	ReF	MRMR	JMI	U	M	IG	ReF	MRMR	JMI	U	M	IG	ReF	MRMR	JMI	U	M
Some nafta burning		✔	✔		↑	↑*		✔	✔		↑*	↑*		✔	✔		↑			✔	✔		↑*	
Frequent leaves burning		✔	✔					✔	✔		↑			✔	✔					✔	✔			
Frequent nafta burning		✔	✔					✔	✔					✔	✔					✔	✔			↑*
Positive cockroach skin prick test		✔	✔					✔	✔					✔	✔					✔	✔			
Father with wheeze		✔	✔					✔	✔		↑*	↑*		✔	✔		↑*			✔	✔			
Child with asthma in last year		✔	✔					✔	✔					✔	✔					✔	✔			
Water from river/stream		✔	✔					✔	✔					✔	✔					✔	✔			
Household has thatched roof		✔	✔					✔	✔					✔	✔					✔	✔			
Application of dung to farm fields		✔	✔					✔	✔		↑*			✔	✔					✔	✔			
Father with hay fever		✔	✔			↑*		✔	✔					✔	✔					✔	✔			↑*
Family size greater than 9		✔	✔					✔	✔					✔	✔		↑*	↑*		✔	✔			
Mother with hay fever		✔	✔					✔	✔					✔	✔					✔	✔			
Dust mite skin prick test		✔	✔					✔	✔		↑*			✔	✔		↑*	↑*		✔	✔			
Frequent gas burning		✔	✔		↑*			✔	✔					✔	✔					✔	✔			
Platelets’ count			✔	✔					✔	✔					✔	✔					✔	✔		
Have a pig		✔	✔					✔	✔					✔	✔					✔	✔			
Urban Residence				✔	↓	↓*				✔	↓					✔	↓					✔		
High lymphocytes count		✔	✔		↑*									✔	✔					✔	✔			
Cooking inside living area					↓*		✔		✔	✔	↓	↓*				✔	↓							
Some leaves burning							✔			✔	↑					✔	↑							
Frequent dung burning		✔	✔					✔												✔	✔			
Mean corpuscular volume	✔			✔						✔						✔						✔		
White blood cell count				✔						✔	↑					✔						✔		
Hemoglobin				✔						✔						✔						✔		
Some gas burning					↑*					✔	↑				✔		↑						↑*	
Mean corpuscular hemoglobin				✔						✔						✔						✔		
Hematocrit				✔						✔						✔	↑*	↑*				✔		↑*
Some wood burning				✔						✔	↑*					✔	↑*					✔		
Red blood cell count				✔						✔	↑*					✔						✔		
Mother with wheeze		✔						✔						✔						✔				
Mean corpuscular hemoglobin concentration				✔	↑*					✔						✔						✔		
Father with asthma		✔						✔						✔						✔				

### Classification performance

**Fig 1:**
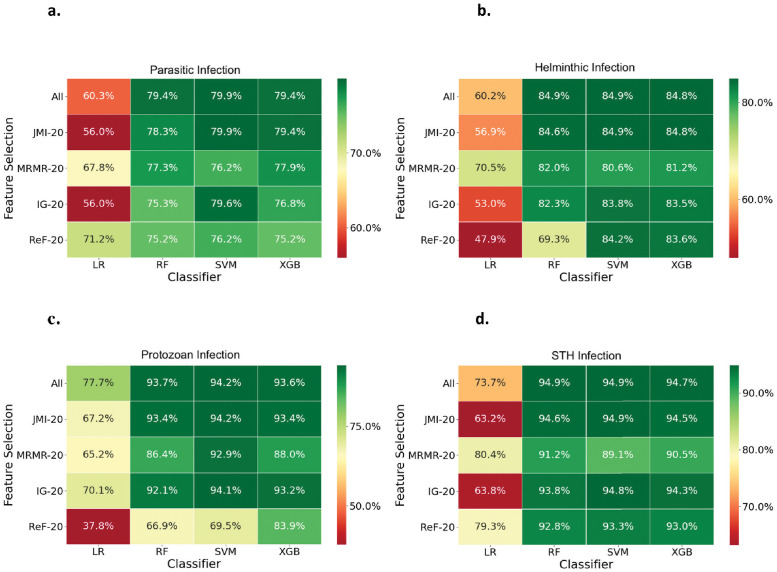
Heatmaps illustrating the accuracy scores for different feature selection and classifier combinations using SMOTE; for infection by (a) any parasite, (b) any helminth, (c) protozoan, and (d) any STH. Green indicates a high accuracy, while red indicates a low accuracy. Feature selection include all features, or top 20 features selected through Joint Mutual Information (JMI-20), Minimum Redundancy Maximum Relevance (MRMR-20), InfoGain (IG-20) and ReliefF (ReF-20). Classifiers include Logistic Regression (LR), Random Forests (RF), Support Vector Machines (SVM), and XGBoost (XGB).

The RF and SVM classifiers had the highest predictive accuracy for all infection outcomes. Using SVM and JMI, we obtained an accuracy of 79.9% for predicting whether a child has an infectious parasite or not (Fig 1A). Additionally, using SVM and ReF, we obtained the highest accuracy of 84.9% for predicting whether a child has or does not have a helminth infection (Fig 1B) The RF and SVM models predicted protozoan and STH infection status with the highest accuracy (94.2% and 94.9%, respectively) (Fig 1C and 1D). These accuracies were obtained primarily by utilizing all risk factors or by utilizing a subset of risk factors. SVM, RF, and XGB achieved comparable results across all infection outcomes, and their accuracy was higher than Logistic Regression (LR). Similarly, on average, all feature selection methods were equally accurate. However, combining JMI feature selection with SVM classification resulted in the highest overall accuracy across four infection outcomes (Fig 1) We also observed that risk factors selected through feature selection methods lead to similar prediction accuracy than classification using all features. Classification with data balancing using SMOTE produced comparable accuracies to classification without any data balancing. However, LR’s classification accuracy was significantly worse with SMOTE than without any data balancing. (Fig 1 and S1 Fig).

**Fig 2:**
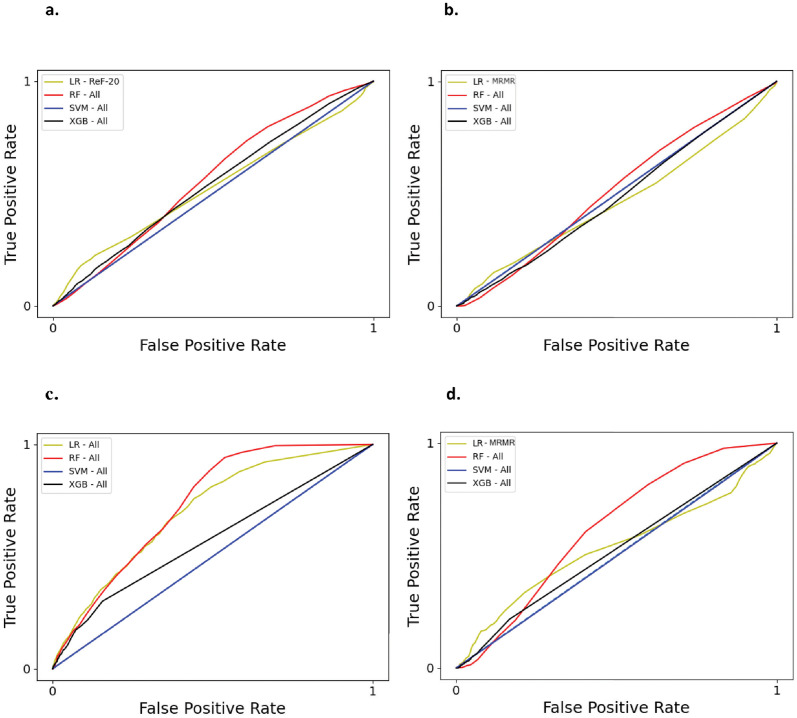
Receiver operating characteristic (ROC) curves for different classifiers using best feature selection method and best hyperparameters for that infection outcome with SMOTE; for infection by (a) any parasite, (b) any helminth, (c) protozoan, and (d) any STH. Classifiers include Logistic Regression (LR), Random Forests (RF), Support Vector Machines (SVM), and XGBoost (XGB). Blue dashed line represents the ROC curve for a random guess. The Area Under Curve (AUC) scores are (a) LR: 0.52, RF: 0.56, SVM: 0.50, XGB: 0.54, (b) LR: 0.47, RF: 0.51, SVM: 0.50, XGB: 0.48, (c) LR: 0.70, RF: 0.73, SVM: 0.50, XGB: 0.58, and (d) LR: 0.54, RF: 0.62, SVM: 0.50, XGB: 0.52.

We created receiver operating characteristic (ROC) curves and calculated area under the ROC curves (AUC) to assess the performance of classification methods using the set of features and hyperparameters that produced the highest accuracy scores. Across all parasitic infections, we found that RF had higher AUC scores than SVM, XGB, and LR (Fig 2). Data balancing slightly decreased the AUC scores for LR and XGB, though marginally increased for RF (S2 Fig).

### Comparative performance of feature selection methods

We observed a distinction between the most frequently occurring risk factors within ranking-based (IG and ReF) and subset-based (JMI and MRMR) techniques. IG and ReF did not frequently identify the same features, with the exception of the protozoan infection. As for subset-based methods, there was very little overlap in the selected features, except for platelets count, which both JMI and MRMR selected for all four infection outcomes. The greatest overlap was observed between ReF and MRMR across all four outcomes (Table 2).

In terms of robustness, both ranking and subset-based methods tended to identify the top features, despite the high cutoff of 95%. Most of the top features were identified by ReF and MRMR simultaneously, while IG and JMI showed relatively less robust and identified few features across the infection outcomes (Table 2).

### Association rules

We found some overlap between the features discovered through feature selection, logistic regression, and association rule learning. Due to a large number of rules with tied lift and confidence values, we chose five association rules for each outcome with risk factors that appeared in at least 20% of the top rules. This way, we avoided choosing similar and redundant association rules. The key results for the association rules can be found in Table 3. Burning of nafta, wood, charcoal, and dung were found in 10 of the 20 rules across the four outcomes, as well as in feature selection (Table 3). Additionally, a strong association was discovered between owning dogs and horses and having parasitic infections. However, neither of these animals was found to be significant in logistic regression models for parasite infection. Additionally, certain rules identified sex as a significant associative predictor, which was not detected in any of the other analyses conducted in the study. Similar to feature selection, association rules showed a combination of different hematological parameters, such as a low or high mean corpuscular hemoglobin concentration and low white blood cell count, to be significant predictors. Additionally, urban residence was found in two association rules for STH infection, despite our findings from logistic regression models indicating that urban residence is protective against STH infection. Finally, association rule learning also found deworming to be positively associated with protozoal and helminthic infections.

**Table 3:** The top five rules based on association rule learning with SMOTE for each infection outcome. For each infection, the five rules with the highest lift values are chosen and sorted. The combinations of risk factors specified on the left leads to the given infection.

**Parasitic Infection**	Support	Confidence	Lift
Child’s sleeping, Smokers in the household, Sex, Family of size 6 to 9 members	0.011	1	2
Ages between six to ten, Water from open well, Waste disposal in open pit, Low White Blood cell count	0.011	1	2
Have a dog, Have a horse, Frequent wood burning, Low mean corpuscular hemoglobin concentration	0.010	1	2
Have a horse, Low hemoglobin, Sex, Low mean corpuscular hemoglobin concentration	0.010	1	2
Have a horse, Sex, Frequent wood burning, Low mean corpuscular hemoglobin concentration	0.010	1	2
**STH Infection**	Support	Confidence	Lift
Urban residence, Have a cow, Frequent nafta burning, Some wood burning	0.014	1	2
Informal maternal education, Some dung burning, Some nafta burning, Low mean corpuscular volume	0.014	1	2
Low hemoglobin, Some charcoal burning, Frequent nafta burning, Some wood burning	0.014	1	2
Urban residence, Informal maternal education, Frequent nafta burning, Some wood burning	0.014	1	2
Informal maternal education, Some charcoal burning, Frequent nafta burning, Some wood burning	0.014	1	2
**Protozoan Infection**	Support	Confidence	Lift
Sex, Low mean corpuscular hemoglobin, High mean corpuscular hemoglobin concentration, Low mean corpuscular volume	0.016	1	2
Dewormed, Some wood burning, High mean corpuscular hemoglobin concentration, Low mean corpuscular volume	0.016	1	2
Dewormed, Sex, High mean corpuscular hemoglobin concentration, Low mean corpuscular volume	0.016	1	2
Dewormed, Low hemoglobin, High mean corpuscular hemoglobin concentration, Low mean corpuscular volume	0.016	1	2
Some gas burning, Some wood burning, Mother is a housewife, Low White Blood cell count	0.016	1	2
**Helminthic Infection**	Support	Confidence	Lift
Have a sheep, Frequent charcoal burning, Low mean corpuscular hemoglobin, Low mean corpuscular volume	0.013	1	2
Have a sheep, Sex, Frequent charcoal burning, Low mean corpuscular hemoglobin	0.011	1	2
Child’s mattress, Child’s sleeping, Cooking in living area, Age greater than 10	0.009	1	2
Child’s bed, Child’s mattress, Cooking in living area, Age greater than 10	0.009	1	2
Child’s mattress, Child’s sleeping, Dewormed, Age greater than 10	0.009	1	2

## Discussion

In our study, we used a variety of machine learning approaches to identify risk factors for four types of parasitic infection as outcome variables. According to several previous studies, machine learning classifiers do not outperform logistic regression in predictive tasks [40–42]. Here, we demonstrated that the SVM, RF and XGB achieved the higher accuracy than LR for all four infection outcomes (Fig 1) Our highest accuracies were obtained when features from JMI were used, indicating that selected features may contain useful information for predictive purposes.

We demonstrated that data balancing with SMOTE did not improve classification accuracy for all infection outcomes in our analysis. This may be due to the rarity of infections, necessitating a significant oversampling of cases in order to balance the data set. This may have resulted in the removal of a substantial amount of variation from our data set, preventing us from achieving a higher level of accuracy.

We found a strong correlation between lower hematological parameters and parasitic infections using feature selection methods and association rules. Previous research indicates that this association is due to intestinal parasites associated with appetite loss and malnutrition, which can manifest as symptoms of anemia in children [43]. We also found association between intestinal parasites and burning of fuels like leaves, nafta, wood, charcoal, and dung. This could be due to the correlation between the use of such fuels, as opposed to electricity as a proxy of lower socioeconomic status. Alternatively compared to nafta uses, study subjects who use wood as main source of fuel are farmers and are usually exposed to soil without protective shoe that could increase chance of acquiring hookworm and other STH infection. A previous study in Ethiopia also confirms that there is a higher prevalence of STHs among farmers than others. [44]

Additionally, we discovered that living in an urban area and cooking in the living room were protective factors. This may be because these characteristics serve as proxy indicators of higher socioeconomic status. Previous research has established a link between low socioeconomic status and an increased risk of intestinal parasites [45]. Lastly, our findings that paternal hay fever and wheeze are generally strong predictors of outcome variables may suggest that families with respiratory allergies are also at compromised immune system, with an increased risk susceptibility of parasitic infections [46]. Also, it could be a result of these factors acting as a proxy measure for other respiratory problems in the family or health access. Lack of access to health care has been linked to intestinal parasite infections [47]. However, the relationship between atopic diseases and helminthic infection remains controversial. Some studies showed that decreased prevalence of atopy among helminthic infected [48, 49], while others showed either positive, or no relation between helminthic infection and allergic disease [50, 51]. Most these studies are cross sectional, and do not allow making any strong temporal associations. A more reliable longitudinal study by Cooper et al [52] showed that a deworming programme in Ecuadorian schoolchildren reduced helminthiases without promoting atopy or atopic diseases. Our study population also enrolled in mass deworming program, which limits the possibility that helminthic induced allergic disorder to be an alternative explanation. Furthermore, a positive association between dust mite skin prick test and infection with any parasite in this study could be due to helminths induced non-functional sensitization of IgE against environmental allergens. Doyen et al [53] documented that Helminth induced sensitization to Dpt was not explained by sensitization to N-glycans nor to major allergens.

In this study protozoan infection was inversely associated with child wheezing but not with paternal wheezing. This could partly explained by difference in age of the population being studied and/or the time of infection. Studies showed that early life infection reduced allergic disorders due to the fact that programming of Th cell memory against allergens commonly occurs during early childhood [54]. However, others have reported conflicting findings either no [55] or positive [56] association between protozoan infection and childhood wheezing. Further investigation is required to elucidate immune regulatory mechanisms involved in the association between intestinal protozoan infections and allergic manifestations.

Regarding association rule learning, we discovered that deworming in conjunction with anemia was positively associated with protozoan infections. However, deworming was only found to have a mitigative effect for STH infections in our multivariate logistic regressions, which is supported by previous literature [57]. This may suggest that deworming may be creating an environment for other intestinal parasites such as protozoans by mitigating STH infections, as some previous studies have suggested [19]. In association rule learning, we also observed that having animals such as dogs, cows, horses, and sheep was positively associated with different intestinal parasites, which agrees with previous studies that have found a higher prevalence of intestinal parasitosis among children raising animals compared to those not raising animals [58]. This association may be because raising animals may act as a proxy for rural residence, but could also be a result of fecal contamination by infected animals. Our analysis found urban residence in two association rules as increased risk for STH infection, despite conflicting in logistic regression models. This discrepancy could be due to differences in these models. Unlike the logistic regression model, which relies on a single model to compute the probability that the module has a fault (i.e., fault-prone) based on its module metrics, association rule mining is that a large set of rules can characterize various types of faulty modules. This could increase the prediction performance since we can select rules based on interestingness measures of a rule such as support and confidence [59]

Our findings provided support for the hypothesis that hematological parameters associated with anemia are strong predictors of intestinal parasitic infections, possibly due to the strong biological connection. Future studies may prioritize data collected from blood samples for predictive purposes due to the high biological relevance of blood samples to intestinal parasites. Our findings, however, demonstrated the importance of socioeconomic and atopy-related factors, implying the importance of combining internal biological parameters with certain external risk factors.

Recent advances in computational capacity and machine learning (ML) have shown the ability to accurately identify patients at high risk of mortality [60] and cardiovascular disease [61] using electronic health record (EHR) data. Although such an approach is not commonly used in parasite epidemiological surveys, our ML algorithm may shed light on new possibilities to use and develop new models to assess risk factors in similar epidemiological studies. Furthermore, applying association rule mining to parasite survey data sets could further confirm existing knowledge regarding parasite risk factors and discover new risk factors that could potentially lead to improved prevention efforts, decision support, and hypothesis generation. However, further clinical and biomedical studies should be done to determine the clinical validity of the new associations generated in our machine learning algorithm.

### Limitations

Our findings should be considered in light of some limitations. First, this is a cross-sectional data collection which makes it difficult to attribute causality based on the observed association. Second, the size of our data set and the class imbalance in our data limited our model’s predictive capabilities. Additionally, by oversampling certain combinations of features using SMOTE, we may have increased the bias in our association rule learning. Third, it is worth noting that while machine learning methods can help us understand the significance of certain risk factors, they do not always indicate whether these risk factors are additive or mitigating. As a result, it is best to combine traditional statistical techniques with advanced machine learning techniques to obtain a more holistic view of risk factors. Finally, our models may not generalize well to other populations, given that they were trained on data from school children enrolled in one region in Ethiopia. Further validation of these models is recommended in other geographic settings to determine generalizability.

## Conclusion

Our findings emphasize the importance of using machine learning algorithms to identify novel risk factors and validate the significance of previously identified risk factors. While feature selection approaches overlapped with logistic regression in our study, they also revealed a large number of risk factors that were not discovered by these approaches. Additionally, association rule learning revealed links between certain risk factor combinations and parasitic infections that were not evident using logistic regression models or feature selection approaches. Finally, SVM, RF and XGB classifiers produced highly accurate predictive models in comparison to LR classifiers. Our findings demonstrate the importance of combining biologically relevant predictors, such as hematological characteristics, with socioeconomic and health-related factors for predicting parasitic infections.

## Supporting information

S1 Fig Heatmaps illustrating the accuracy scores for different feature selection and classifier combinations without any data balancing; for infection by (a) any parasite, (b) any helminth, (c) protozoan, and (d) STH. Green indicates a high accuracy, while red indicates a low accuracy. Feature selection include All features, or top 20 features selected through Joint Mutual Information (JMI-20), Minimum Redundancy Maximum Relevance (MRMR-20), InfoGain (IG-20) and ReliefF (ReF-20). Classifiers include Logistic Regression (LR), Random Forests (RF), Support Vector Machines (SVM), and XGBoost (XGB).
(TIF)

S2 Fig Receiver operating characteristic (ROC) curves for different classifiers using best feature selection method and best hyperparameters for that infection outcome without data balancing; for infection by (a) any parasite, (b) any helminth, (c) protozoan, and (d) STH. Classifiers include Logistic Regression (LR), Random Forests (RF), Support Vector Machines (SVM), and XGBoost (XGB). Blue dashed line represents the ROC curve for a random guess. The Area Under Curve (AUC) scores are (a) LR: 0.57, RF: 0.57, SVM: 0.55, XGB: 0.53, (b) LR: 0.54, RF: 0.54, SVM: 0.54, XGB: 0.49, (c) LR: 0.72, RF: 0.72, SVM: 0.49, XGB: 0.69, and (d) LR: 0.54, RF: 0.61, SVM: 0.50, XGB: 0.59.
(TIFF)

S1 Table Univariate and multivariate logistic regression analysis of risk factors for STH infection. For each risk factor, corresponding references and survey results are provided. Adjusted p-values are provided in parenthesis.
(DOCX)

S2 Table Univariate and multivariate logistic regression analysis of risk factors for infection with any protozoan. For each risk factor, corresponding references and survey results are provided. Adjusted p-values are provided in parenthesis.
(DOCX)

S3 Table Univariate and multivariate logistic regression analysis of risk factors for infection with any helminth. For each risk factor, corresponding references and survey results are provided. Adjusted p-values are provided in parenthesis.
(DOCX)

S4 Table Univariate and multivariate logistic regression analysis of risk factors for infection with parasites. For each risk factor, corresponding references and survey results are provided. Adjusted p-values are provided in parenthesis.
(DOCX)
