# Supplementary figures and images for: Machine learning-based risk factor analysis and prevalence prediction of intestinal parasitic infections using epidemiological survey data
Source: PLoS Negl Trop Dis. 2022 Jun 14;16(6):e0010517. doi: 10.1371/journal.pntd.0010517 (PMC9236253; doi:10.1371/journal.pntd.0010517)

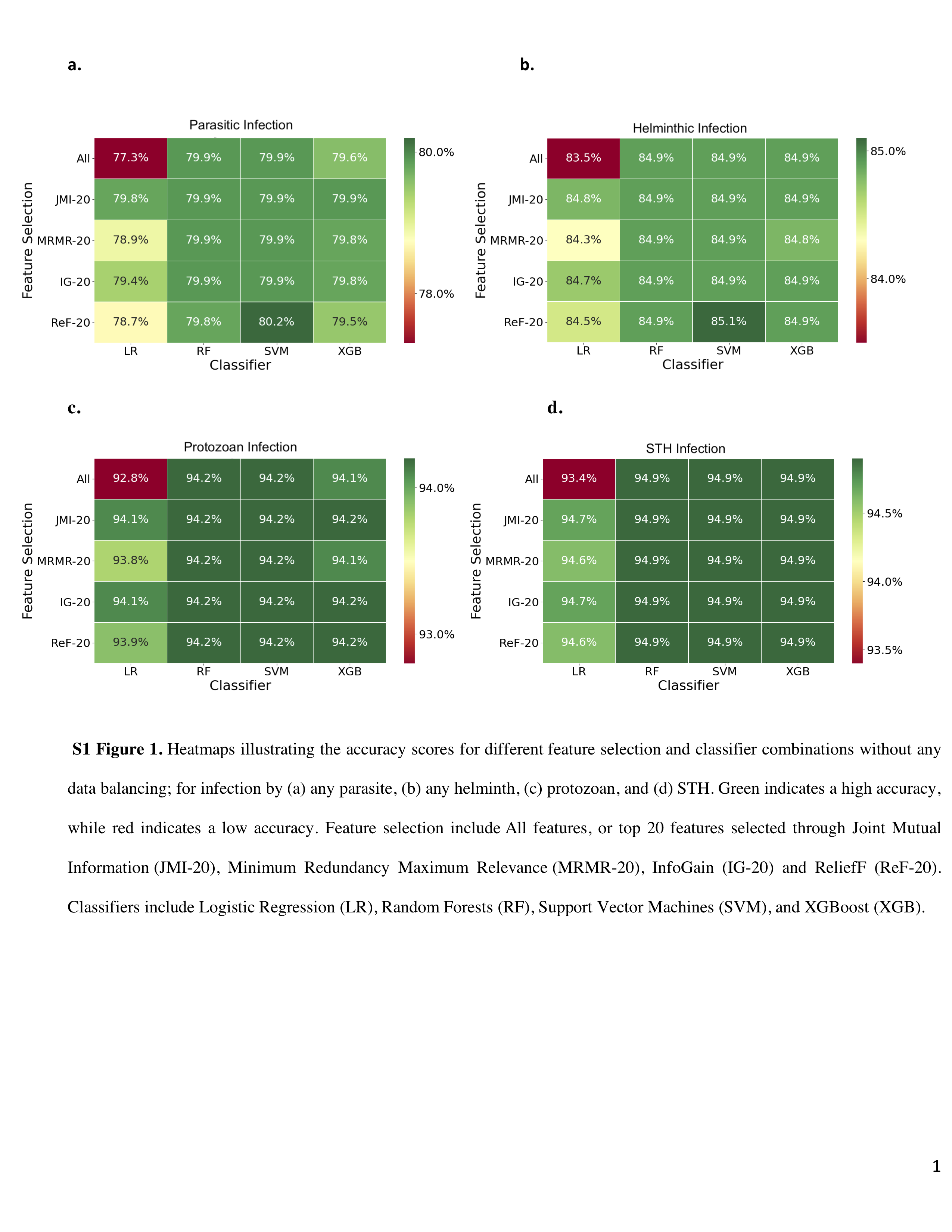

Supplement: S1 Fig — Green indicates a high accuracy, while red indicates a low accuracy. Feature selection include All features, or top 20 features selected through Joint Mutual Information (JMI-20), Minimum Redundancy Maximum Relevance (MRMR-20), InfoGain (IG-20) and ReliefF (ReF-20). Classifiers include Logistic Regression (LR), Random Forests (RF), Support Vector Machines (SVM), and XGBoost (XGB). (TIF) [file pntd.0010517.s001.tif]

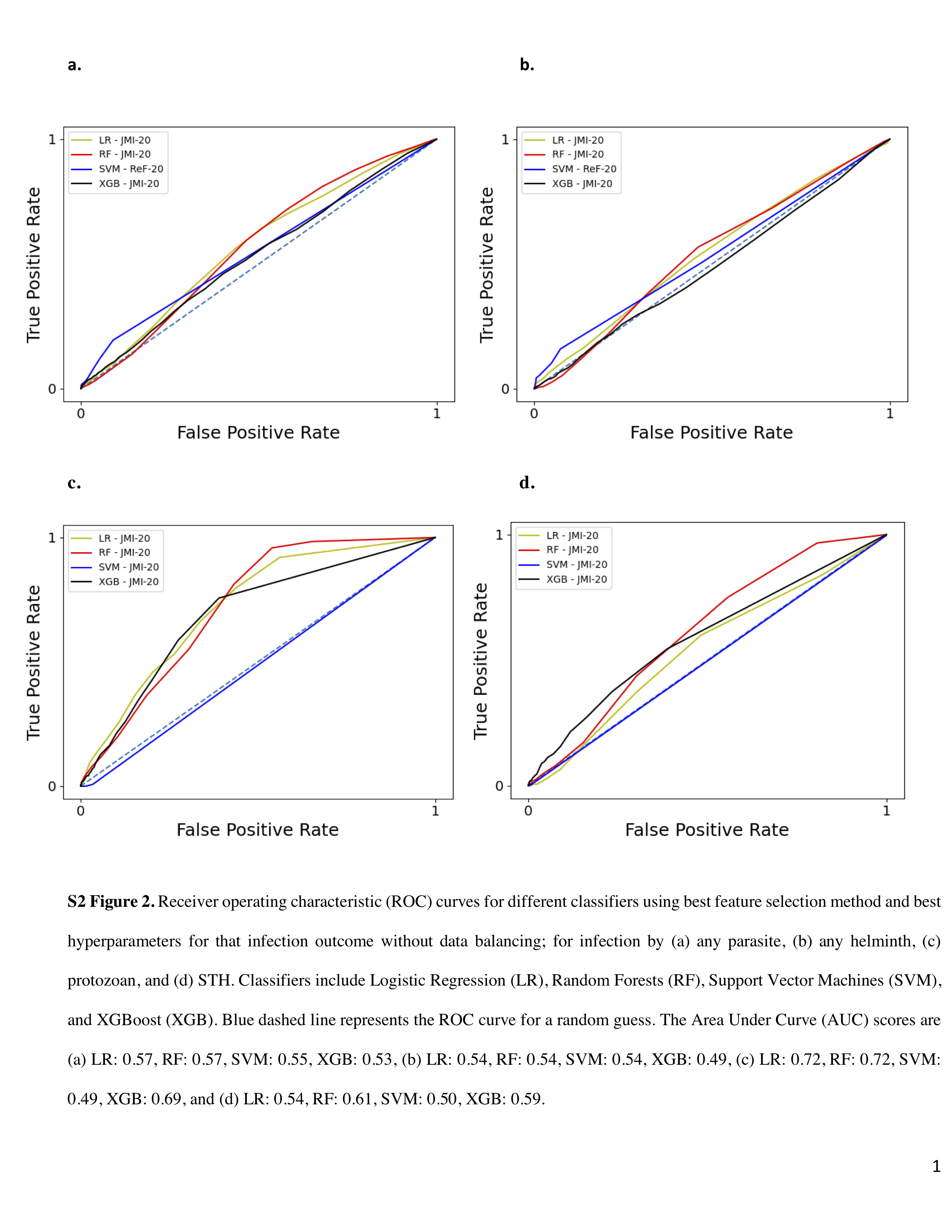

Supplement: S2 Fig — Classifiers include Logistic Regression (LR), Random Forests (RF), Support Vector Machines (SVM), and XGBoost (XGB). Blue dashed line represents the ROC curve for a random guess. The Area Under Curve (AUC) scores are (a) LR: 0.57, RF: 0.57, SVM: 0.55, XGB: 0.53, (b) LR: 0.54, RF: 0.54, SVM: 0.54, XGB: 0.49, (c) LR: 0.72, RF: 0.72, SVM: 0.49, XGB: 0.69, and (d) LR: 0.54, RF: 0.61, SVM: 0.50, XGB: 0.59. (TIFF) [file pntd.0010517.s002.tiff]
