# Supplementary material for: Machine learning-based risk factor analysis and prevalence prediction of intestinal parasitic infections using epidemiological survey data
Source: PLoS Negl Trop Dis. 2022 Jun 14;16(6):e0010517. doi: 10.1371/journal.pntd.0010517 (PMC9236253; doi:10.1371/journal.pntd.0010517)
Supplement: S1 Table — For each risk factor, corresponding references and survey results are provided. Adjusted p-values are provided in parenthesis. (DOCX) [file pntd.0010517.s003.docx]

**S1 Table.** Univariate and multivariate logistic regression analysis of risk factors for STH infection. For each risk factor, corresponding references and survey results are provided. Adjusted p-values are provided in parenthesis.

| **Variable Name** | **Meaning** | **STH (+)** | **STH (-)** | **P value (uni)** | **COR** | **CI-95% (uni)** | **P value (multi)** | **AOR** | **CI-95% (multi)** |
| --- | --- | --- | --- | --- | --- | --- | --- | --- | --- |
| **DEMOGRAPHIC FACTORS** | | | | | | | | | |
| Age |  |  |  |  |  |  |  |  |  |
| 0 | >10 years old |  |  |  |  |  |  |  |  |
| 1 | 6 to 10 years old | 17 (4.2%) | 391 (95.8%) | 0.136 (0.545) | 0.6267 | 0.3326-1.1461 | 0.2422 (0.947) | 0.6233 | 0.2772-1.3657 |
| 2 | <6 years old | 2 (2.3%) | 85 (97.7%) | 0.1443 (0.545) | 0.3391 | 0.0542-1.1549 | 0.4473 (0.97) | 0.4994 | 0.0574-2.4089 |
| Deworming | |  |  |  |  |  |  |  |  |
| 0 | Not dewormed | |  |  |  |  |  |  |  |
| 1 | Dewormed | 33 (4.4%) | 719 (95.6%) | 0.0528 (0.43) | 0.5355 | 0.2895-1.0346 | 0.0277 (0.576) | 0.4174 | 0.1939-0.9293 |
| Family Size | |  |  |  |  |  |  |  |  |
| 0 | <6 people |  |  |  |  |  |  |  |  |
| 1 | 6 to 9 people | 8 (2.6%) | 294 (97.4%) | 0.0269 (0.261) | 0.4168 | 0.1785-0.8592 | 0.049 (0.666) | 0.4094 | 0.1577-0.9519 |
| 2 | >9 people | 2 (10%) | 18 (90%) | 0.4865 (0.862) | 1.7018 | 0.2644-6.1965 | 0.5675 (0.991) | 1.7168 | 0.1969-9.1849 |
| Residence | |  |  |  |  |  |  |  |  |
| 0 | Rural |  |  |  |  |  |  |  |  |
| 1 | Urban | 15 (2.9%) | 511 (97.1%) | 0.0007 (0.034) | 0.3407 | 0.1775-0.625 | 0.0339 (0.576) | 0.3667 | 0.1381-0.8923 |
| Sex |  |  |  |  |  |  |  |  |  |
| 0 | Male |  |  |  |  |  |  |  |  |
| 1 | Female | 29 (5.6%) | 489 (94.4%) | 0.4387 (0.857) | 1.2641 | 0.7036-2.3225 | 0.6047 (0.991) | 1.2077 | 0.5937-2.4999 |
| **SOCIOECONOMIC FACTORS** | | | | | | | | | |
| Sleeps on a bed |  |  |  |  |  |  |  |  |  |
| 0 | No |  |  |  |  |  |  |  |  |
| 1 | Yes | 15 (4.6%) | 313 (95.4%) | 0.5945 (0.962) | 0.8437 | 0.4393-1.5497 | 0.9211 (0.995) | 1.0703 | 0.282-4.0946 |
| Household burns charcoal | |  |  |  |  |  |  |  |  |
| 0 | Never |  |  |  |  |  |  |  |  |
| 1 | Sometimes | 23 (6.6%) | 326 (93.4%) | 0.3616 (0.82) | 1.658 | 0.619-5.7587 | 0.99 (0.995) | 1.0098 | 0.2426-5.3951 |
| 2 | Always | 21 (4.2%) | 474 (95.8%) | 0.9423 (0.986) | 1.0411 | 0.3856-3.63 | 0.9128 (0.995) | 1.0909 | 0.2539-6.0565 |
| Household burns dung | |  |  |  |  |  |  |  |  |
| 0 | Never |  |  |  |  |  |  |  |  |
| 1 | Sometimes | 17 (6.7%) | 235 (93.3%) | 0.2026 (0.599) | 1.4902 | 0.7911-2.7219 | 0.6551 (0.991) | 0.8234 | 0.3439-1.9132 |
| 2 | Always | 1 (2.4%) | 41 (97.6%) | 0.5037 (0.862) | 0.5024 | 0.0279-2.4395 | 0.6602 (0.991) | 0.5858 | 0.0241-4.08 |
| Household burns gas | |  |  |  |  |  |  |  |  |
| 0 | Never |  |  |  |  |  |  |  |  |
| 1 | Sometimes | 10 (10%) | 90 (90%) | 0.0159 (0.216) | 2.466 | 1.1264-4.9612 | 0.6849 (0.991) | 1.2073 | 0.4621-2.898 |
| 2 | Always | 2 (28.6%) | 5 (71.4%) | 0.0105 (0.179) | 8.8778 | 1.2424-42.7566 | 0.3164 (0.964) | 3.8984 | 0.2019-46.8213 |
| Household burns leaves | |  |  |  |  |  |  |  |  |
| 0 | Never |  |  |  |  |  |  |  |  |
| 1 | Sometimes | 20 (6.1%) | 308 (93.9%) | 0.2449 (0.666) | 1.4312 | 0.7743-2.6134 | 0.4994 (0.97) | 0.734 | 0.2981-1.8117 |
| 2 | Always | 3 (7.9%) | 35 (92.1%) | 0.3167 (0.769) | 1.8891 | 0.4351-5.7302 | 0.9041 (0.995) | 0.9057 | 0.1501-4.0584 |
| Household burns nafta | |  |  |  |  |  |  |  |  |
| 0 | Never |  |  |  |  |  |  |  |  |
| 1 | Sometimes | 6 (18.8%) | 26 (81.2%) | 0.001 (0.034) | 4.863 | 1.7352-11.772 | 0.0117 (0.576) | 5.2454 | 1.379-18.6812 |
| 2 | Always | 1 (20%) | 4 (80%) | 0.1412 (0.545) | 5.2683 | 0.2661-36.6089 | 0.0886 (0.861) | 31.6664 | 0.4931-2209.9624 |
| Household burns wood | |  |  |  |  |  |  |  |  |
| 0 | Never |  |  |  |  |  |  |  |  |
| 1 | Sometimes | 26 (5.9%) | 417 (94.1%) | 0.695 (0.965) | 1.1769 | 0.544-2.8325 | 0.4699 (0.97) | 0.6452 | 0.2033-2.2375 |
| 2 | Always | 14 (4.1%) | 326 (95.9%) | 0.6436 (0.965) | 0.8106 | 0.3397-2.0676 | 0.8423 (0.995) | 0.8778 | 0.2479-3.304 |
| Household uses electricity | |  |  |  |  |  |  |  |  |
| 0 | Never |  |  |  |  |  |  |  |  |
| 1 | Sometimes | 6 (6.8%) | 82 (93.2%) | 0.4124 (0.857) | 1.4568 | 0.5368-3.3486 | 0.6992 (0.991) | 0.8017 | 0.24-2.3314 |
| 2 | Always | 9 (5.5%) | 155 (94.5%) | 0.7076 (0.965) | 1.156 | 0.5111-2.3677 | 0.4682 (0.97) | 0.6608 | 0.2048-1.9479 |
| Composition of floor in the home |  |  |  |  |  |  |  |  |  |
| 0 | Any flooring |  |  |  |  |  |  |  |  |
| 1 | Mud | 21 (4.2%) | 484 (95.8%) | 0.1623 (0.555) | 0.6589 | 0.3633-1.1795 | 0.3904 (0.97) | 0.7022 | 0.3103-1.5723 |
| Maternal Education | |  |  |  |  |  |  |  |  |
| 0 | Formal |  |  |  |  |  |  |  |  |
| 1 | Informal | 29 (5.3%) | 517 (94.7%) | 0.7237 (0.965) | 1.113 | 0.6193-2.0453 | 0.5698 (0.991) | 0.8003 | 0.3724-1.7457 |
| Maternal Occupation | |  |  |  |  |  |  |  |  |
| 0 | Professional Employment | |  |  |  |  |  |  |  |
| 1 | Housewife | 26 (5.3%) | 463 (94.7%) | 0.8042 (0.986) | 0.9279 | 0.5145-1.6923 | 0.4089 (0.97) | 1.3712 | 0.6525-2.9465 |
| 2 | Farming | 1 (1.2%) | 84 (98.8%) | 0.1147 (0.545) | 0.1967 | 0.0109-0.9606 | 0.387 (0.97) | 0.3404 | 0.0124-2.5725 |
| Child’s mattress | |  |  |  |  |  |  |  |  |
| 0 | Any mattress |  |  |  |  |  |  |  |  |
| 1 | Grass or no mattress | 13 (7%) | 174 (93%) | 0.2003 (0.599) | 1.5369 | 0.7691-2.8975 | 0.0857 (0.861) | 2.326 | 0.8725-6.0668 |
| Roof on the home |  |  |  |  |  |  |  |  |  |
| 0 | Any roofing except thatched |  |  |  |  |  |  |  |  |
| 1 | Thatched roof | 0 (0%) | 21 (100%) | 0.9864 (0.986) | 0 | 0-Inf | 0.9878 (0.995) | 0 | 0-Inf |
| Composition of walls in the home |  |  |  |  |  |  |  |  |  |
| 0 | Cement, brick, or iron | |  |  |  |  |  |  |  |
| 1 | Wood or grass | 33 (5%) | 631 (95%) | 0.7864 (0.986) | 0.917 | 0.4985-1.7635 | 0.1388 (0.94) | 0.5094 | 0.2075-1.2535 |
| What the child sleeps on | | |  |  |  |  |  |  |  |
| 0 | Bed |  |  |  |  |  |  |  |  |
| 1 | Floor | 16 (4.9%) | 309 (95.1%) | 0.8613 (0.986) | 0.9466 | 0.4995-1.7265 | 0.8149 (0.995) | 0.855 | 0.226-3.0771 |
| **HEALTH FACTORS** | | | | | | | | | |
| Cockroach skin prick test | | |  |  |  |  |  |  |  |
| 0 | Negative |  |  |  |  |  |  |  |  |
| 1 | Positive | 2 (9.1%) | 20 (90.9%) | 0.3964 (0.857) | 1.9 | 0.2977-6.781 | 0.7557 (0.995) | 1.3795 | 0.1351-8.7313 |
| Child has asthma | |  |  |  |  |  |  |  |  |
| 0 | No |  |  |  |  |  |  |  |  |
| 1 | Yes | 1 (3.2%) | 30 (96.8%) | 0.6336 (0.965) | 0.6128 | 0.034-2.9585 | 0.2934 (0.964) | 0.2932 | 0.0138-1.976 |
| Child has hay fever | |  |  |  |  |  |  |  |  |
| 0 | No |  |  |  |  |  |  |  |  |
| 1 | Yes | 3 (4.2%) | 69 (95.8%) | 0.7097 (0.965) | 0.7971 | 0.1901-2.2549 | 0.5302 (0.991) | 0.4893 | 0.0451-4.0195 |
| Child has had hay fever in last year | | | |  |  |  |  |  |  |
| 0 | No |  |  |  |  |  |  |  |  |
| 1 | Yes | 4 (5%) | 76 (95%) | 0.9676 (0.986) | 0.9785 | 0.289-2.4958 | 0.7937 (0.995) | 0.7602 | 0.0812-4.9884 |
| Child with rash in last year | | |  |  |  |  |  |  |  |
| 0 | No |  |  |  |  |  |  |  |  |
| 1 | Yes | 9 (5.4%) | 159 (94.6%) | 0.8649 (0.986) | 1.0668 | 0.4764-2.1506 | 0.7873 (0.995) | 0.876 | 0.3125-2.1851 |
| Child has wheeze in last year | | |  |  |  |  |  |  |  |
| 0 | No |  |  |  |  |  |  |  |  |
| 1 | Yes | 7 (9.3%) | 68 (90.7%) | 0.0883 (0.545) | 2.0739 | 0.8256-4.5301 | 0.1797 (0.94) | 2.2275 | 0.6466-6.8838 |
| Dust mite skin prick test | |  |  |  |  |  |  |  |  |
| 0 | Negative |  |  |  |  |  |  |  |  |
| 1 | Positive | 2 (14.3)% | 12 (85.7)% | 0.1357 (0.545) | 3.1957 | 0.4882-12.1723 | 0.1167 (0.94) | 4.9842 | 0.5173-33.2954 |
| Father with asthma | |  |  |  |  |  |  |  |  |
| 0 | No |  |  |  |  |  |  |  |  |
| 1 | Yes | 2 (6.1%) | 31 (93.9%) | 0.7978 (0.986) | 1.2104 | 0.1923-4.1724 | 0.5927 (0.991) | 0.4498 | 0.0189-6.1643 |
| Father with hay fever | | |  |  |  |  |  |  |  |
| 0 | No |  |  |  |  |  |  |  |  |
| 1 | Yes | 2 (12.5%) | 14 (87.5%) | 0.1922 (0.599) | 2.7329 | 0.4212-10.1713 | 0.0226 (0.576) | 12.3064 | 1.1396-97.6567 |
| Father with wheeze | |  |  |  |  |  |  |  |  |
| 0 | No |  |  |  |  |  |  |  |  |
| 1 | Yes | 3 (12.5%) | 21 (87.5%) | 0.1089 (0.545) | 2.7714 | 0.6381-8.4245 | 0.4792 (0.97) | 2.4601 | 0.1703-26.6437 |
| Mother with asthma | |  |  |  |  |  |  |  |  |
| 0 | No |  |  |  |  |  |  |  |  |
| 1 | Yes | 4 (9.1%) | 40 (90.9%) | 0.2251 (0.638) | 1.9409 | 0.5645-5.0907 | 0.2389 (0.947) | 2.5312 | 0.4591-10.7535 |
| Mother with hay fever | | |  |  |  |  |  |  |  |
| 0 | No |  |  |  |  |  |  |  |  |
| 1 | Yes | 0 (0%) | 16 (100%) | 0.9818 (0.986) | 0 | 0-Inf | 0.9903 (0.995) | 0 | 0-Inf |
| Mother with wheeze | |  |  |  |  |  |  |  |  |
| 0 | No |  |  |  |  |  |  |  |  |
| 1 | Yes | 1 (3.6%) | 27 (96.4%) | 0.7112 (0.965) | 0.6832 | 0.0379-3.3185 | 0.8342 (0.995) | 0.7523 | 0.0255-7.2383 |
| **ENVIRONMENTAL FACTORS** | | | | | | | | | |
| Application of dung to farm fields | |  |  |  |  |  |  |  |  |
| 0 | No |  |  |  |  |  |  |  |  |
| 1 | Yes | 1 (5.9%) | 16 (94.1%) | 0.8818 (0.986) | 1.1676 | 0.064-5.9114 | 0.6918 (0.991) | 1.6599 | 0.0682-14.5227 |
| Cigarette smokers in the house | | |  |  |  |  |  |  |  |
| 0 | No |  |  |  |  |  |  |  |  |
| 1 | Yes | 3 (5.2%) | 55 (94.8%) | 0.9781 (0.986) | 1.017 | 0.2415-2.9001 | 0.7477 (0.995) | 1.2581 | 0.2505-4.4523 |
| Location of cooking area | |  |  |  |  |  |  |  |  |
| 0 | Outside living area | |  |  |  |  |  |  |  |
| 1 | Inside living area | 9 (2.5%) | 352 (97.5%) | 0.0059 (0.135) | 0.3553 | 0.1597-0.71 | 0.1754 (0.94) | 0.4513 | 0.1356-1.372 |
| Family has a cat | |  |  |  |  |  |  |  |  |
| 0 | No |  |  |  |  |  |  |  |  |
| 1 | Yes | 13 (4.5%) | 275 (95.5%) | 0.5905 (0.962) | 0.8361 | 0.4204-1.5666 | 0.2508 (0.947) | 0.5922 | 0.2318-1.4027 |
| Family has a cow | |  |  |  |  |  |  |  |  |
| 0 | No |  |  |  |  |  |  |  |  |
| 1 | Yes | 13 (6.4%) | 190 (93.6%) | 0.3403 (0.798) | 1.3762 | 0.6894-2.5907 | 0.3177 (0.964) | 1.6566 | 0.591-4.3393 |
| Family has a dog | |  |  |  |  |  |  |  |  |
| 0 | No |  |  |  |  |  |  |  |  |
| 1 | Yes | 22 (5.7%) | 365 (94.3%) | 0.4929 (0.862) | 1.2263 | 0.6791-2.1958 | 0.4985 (0.97) | 1.3151 | 0.5877-2.8999 |
| Family has a hen | |  |  |  |  |  |  |  |  |
| 0 | No |  |  |  |  |  |  |  |  |
| 1 | Yes | 13 (6%) | 205 (94%) | 0.5071 (0.862) | 1.2484 | 0.6259-2.3473 | 0.9526 (0.995) | 0.9733 | 0.3836-2.315 |
| Family has a horse | |  |  |  |  |  |  |  |  |
| 0 | No |  |  |  |  |  |  |  |  |
| 1 | Yes | 5 (5.2%) | 91 (94.8%) | 0.9577 (0.986) | 1.0261 | 0.3482-2.4289 | 0.6467 (0.991) | 0.7422 | 0.188-2.4876 |
| Family has a pig | |  |  |  |  |  |  |  |  |
| 0 | No |  |  |  |  |  |  |  |  |
| 1 | Yes | 0 (0%) | 5 (100%) | 0.9845 (0.986) | 0 | 0-Inf | 0.9951 (0.995) | 0 | 0-Inf |
| Family has a sheep | |  |  |  |  |  |  |  |  |
| 0 | No |  |  |  |  |  |  |  |  |
| 1 | Yes | 10 (6.3%) | 148 (93.7%) | 0.4409 (0.857) | 1.3265 | 0.6136-2.6219 | 0.8507 (0.995) | 1.108 | 0.3613-3.1105 |
| Source of water in household | |  |  |  |  |  |  |  |  |
| 0 | Piped |  |  |  |  |  |  |  |  |
| 1 | Well | 5 (6.3%) | 74 (93.7%) | 0.6428 (0.965) | 1.2539 | 0.424-2.9874 | 0.8412 (0.995) | 1.129 | 0.3122-3.4659 |
| 2 | River or stream | 0 (0%) | 22 (100%) | 0.9861 (0.986) | 0 | 0-Inf | 0.9892 (0.995) | 0 | 0-Inf |
| Type of toilet in the home | |  |  |  |  |  |  |  |  |
| 0 | Any toilet |  |  |  |  |  |  |  |  |
| 1 | None | 3 (4.1%) | 70 (95.9%) | 0.6906 (0.965) | 0.7848 | 0.1872-2.219 | 0.2452 (0.947) | 0.4167 | 0.0777-1.5965 |
| Location of household waste disposal | |  |  |  |  |  |  |  |  |
| 0 | Garbage Bin |  |  |  |  |  |  |  |  |
| 1 | Open Field | 14 (7.2%) | 180 (92.8%) | 0.1631 (0.555) | 1.6028 | 0.804-3.0603 | 0.3826 (0.97) | 1.4838 | 0.5973-3.5562 |
| 2 | Pit | 6 (4.2%) | 137 (95.8%) | 0.8234 (0.986) | 0.9025 | 0.3323-2.0809 | 0.8373 (0.995) | 0.8927 | 0.2768-2.4964 |
| **HEMATOLOGICAL FACTORS** | | | | | | | | | |
| Hematocrit | |  |  |  |  |  |  |  |  |
| Continuous | | - | - | 0.8289 (0.986) | 0.996 | 0.9434-1.0122 | 0.6164 (0.991) | 0.9504 | 0.769-1.0245 |
| Hemoglobin | |  |  |  |  |  |  |  |  |
| Continuous | | - | - | 0.9653 (0.986) | 0.9961 | 0.8483-1.1798 | 0.4259 (0.97) | 0.846 | 0.5734-1.2782 |
| Lymphocytes’ count | |  |  |  |  |  |  |  |  |
| 0 |  |  |  |  |  |  |  |  |  |
| 1 | Low | 16 (4.3%) | 354 (95.7%) | 0.4964 (0.862) | 0.806 | 0.4233-1.4805 | 0.9948 (0.995) | 1.0027 | 0.4367-2.2554 |
| 2 | High | 2 (28.6%) | 5 (71.4%) | 0.0219 (0.249) | 7.1333 | 0.9934-34.632 | 0.2744 (0.964) | 3.5113 | 0.2864-29.0777 |
| Mean Corpuscular Hemoglobin | | |  |  |  |  |  |  |  |
| Continuous | | - | - | 0.1243 (0.545) | 0.9033 | 0.7993-1.0367 | 0.3262 (0.964) | 0.8592 | 0.6504-1.2154 |
| Mean Corpuscular Hemoglobin Concentration | | | |  |  |  |  |  |  |
| Continuous | | - | - | 0.0568 (0.43) | 1.0962 | 1.0007-1.2584 | 0.1317 (0.94) | 1.1029 | 0.9932-1.3462 |
| Mean Corpuscular Volume | | |  |  |  |  |  |  |  |
| Continuous | | - | - | 0.2604 (0.681) | 0.9793 | 0.9475-1.0214 | 0.1735 (0.94) | 1.1142 | 0.9752-1.2998 |
| Platelet count | |  |  |  |  |  |  |  |  |
| Continuous | | - | - | 0.416 (0.857) | 1.0013 | 0.998-1.0045 | 0.4047 (0.97) | 1.0019 | 0.9975-1.0065 |
| Red Blood Cell count | |  |  |  |  |  |  |  |  |
| Continuous | | - | - | 0.1019 (0.545) | 1.5041 | 0.9-2.3942 | 0.1974 (0.947) | 2.8383 | 0.6375-14.562 |
| White Blood Cell count | | |  |  |  |  |  |  |  |
| Continuous | | - | - | 0.3163 (0.769) | 0.9392 | 0.8257-1.0549 | 0.6106 (0.991) | 0.9603 | 0.8167-1.1178 |
