# Supplementary material for: Machine learning-based risk factor analysis and prevalence prediction of intestinal parasitic infections using epidemiological survey data
Source: PLoS Negl Trop Dis. 2022 Jun 14;16(6):e0010517. doi: 10.1371/journal.pntd.0010517 (PMC9236253; doi:10.1371/journal.pntd.0010517)
Supplement: S2 Table — For each risk factor, corresponding references and survey results are provided. Adjusted p-values are provided in parenthesis. (DOCX) [file pntd.0010517.s004.docx]

**S2 Table.** Univariate and multivariate logistic regression analysis of risk factors for infection with any protozoan. For each risk factor, corresponding references and survey results are provided. Adjusted p-values are provided in parenthesis.

| **Variable Name** | **Meaning** | **Protozoan (+)** | **Protozoan (-)** | **P value (uni)** | **COR** | **CI-95% (uni)** | **P value (multi)** | **AOR** | **CI-95% (multi)** |
| --- | --- | --- | --- | --- | --- | --- | --- | --- | --- |
| **DEMOGRAPHIC FACTORS** | | | | | | | | | |
| Age |  |  |  |  |  |  |  |  |  |
| 0 | >10 |  |  |  |  |  |  |  |  |
| 1 | 6 to 10 | 18 (4.4%) | 390 (95.6%) | 0.041 (0.155) | 0.5433 | 0.2966-0.9629 | 0.4434 (0.891) | 0.7306 | 0.3234-1.6244 |
| 2 | <6 | 2 (2.3%) | 85 (97.7%) | 0.0814 (0.252) | 0.277 | 0.0445-0.9321 | 0.6626 (0.891) | 0.6786 | 0.0881-3.2985 |
| Deworming | |  |  |  |  |  |  |  |  |
| 0 | Not dewormed | |  |  |  |  |  |  |  |
| 1 | Dewormed | 46 (6.1%) | 706 (93.9%) | 0.4697 (0.665) | 1.3104 | 0.6596-2.906 | 0.3809 (0.853) | 1.5313 | 0.6165-4.2226 |
| Family Size | |  |  |  |  |  |  |  |  |
| 0 | <6 |  |  |  |  |  |  |  |  |
| 1 | 6 to 9 | 14 (4.6%) | 288 (95.4%) | 0.2725 (0.463) | 0.7049 | 0.365-1.2859 | 0.6681 (0.891) | 0.838 | 0.3656-1.8572 |
| 2 | >9 | 1 (5%) | 19 (95%) | 0.7947 (0.886) | 0.7632 | 0.0419-3.827 | 0.3884 (0.853) | 0.3606 | 0.0169-2.6223 |
| Residence | |  |  |  |  |  |  |  |  |
| 0 | Rural |  |  |  |  |  |  |  |  |
| 1 | Urban | 16 (3%) | 510 (97%) | 0.0001 (0.002) | 0.3033 | 0.1625-0.5408 | 0.8386 (0.95) | 1.0872 | 0.477-2.4089 |
| Sex |  |  |  |  |  |  |  |  |  |
| 0 | Male |  |  |  |  |  |  |  |  |
| 1 | Female | 36 (6.9%) | 482 (93.1%) | 0.1106 (0.284) | 1.5921 | 0.9098-2.8718 | 0.3885 (0.853) | 1.3625 | 0.6801-2.7972 |
| **SOCIOECONOMIC FACTORS** | | | | | | | | | |
| Bed |  |  |  |  |  |  |  |  |  |
| 0 | No |  |  |  |  |  |  |  |  |
| 1 | Yes | 13 (4%) | 315 (96%) | 0.0763 (0.247) | 0.5621 | 0.2861-1.034 | 0.1867 (0.715) | 0.4114 | 0.1115-1.5774 |
| Household burns charcoal | | |  |  |  |  |  |  |  |
| 0 | Never |  |  |  |  |  |  |  |  |
| 1 | Sometimes | 33 (9.5%) | 316 (90.5%) | 0.0288 (0.123) | 5.0127 | 1.4861-31.2627 | 0.9243 (0.972) | 1.0936 | 0.1963-9.0617 |
| 2 | Always | 20 (4%) | 475 (96%) | 0.3481 (0.551) | 2.0211 | 0.5771-12.7917 | 0.7417 (0.91) | 0.7285 | 0.1254-6.1991 |
| Household burns dung | |  |  |  |  |  |  |  |  |
| 0 | Never |  |  |  |  |  |  |  |  |
| 1 | Sometimes | 25 (9.9%) | 227 (90.1%) | 0.0018 (0.021) | 2.4386 | 1.385-4.2728 | 0.3888 (0.853) | 0.7184 | 0.3338-1.5143 |
| 2 | Always | 2 (4.8%) | 40 (95.2%) | 0.892 (0.963) | 1.1071 | 0.1749-3.8693 | 0.6276 (0.891) | 0.5812 | 0.0428-4.1225 |
| Household burns gas | |  |  |  |  |  |  |  |  |
| 0 | Never |  |  |  |  |  |  |  |  |
| 1 | Sometimes | 16 (16%) | 84 (84%) | 0 (0.001) | 3.8877 | 2.0344-7.1392 | 0.1088 (0.529) | 1.9712 | 0.8407-4.4647 |
| 2 | Always | 0 (0%) | 7 (100%) | 0.9881 (0.988) | 0 | 0-Inf | 0.9943 (0.994) | 0 | 0-Inf |
| Household burns leaves | |  |  |  |  |  |  |  |  |
| 0 | Never |  |  |  |  |  |  |  |  |
| 1 | Sometimes | 40 (12.2%) | 288 (87.8%) | 0 (0) | 7.1338 | 3.7319-14.8006 | 0.009 (0.204) | 3.4589 | 1.4187-9.279 |
| 2 | Always | 4 (10.5%) | 34 (89.5%) | 0.0032 (0.027) | 6.0428 | 1.6094-18.7345 | 0.076 (0.516) | 4.2463 | 0.7917-20.4243 |
| Household burns nafta | |  |  |  |  |  |  |  |  |
| 0 | Never |  |  |  |  |  |  |  |  |
| 1 | Sometimes | 4 (12.5%) | 28 (87.5%) | 0.1152 (0.284) | 2.3922 | 0.6891-6.3895 | 0.8599 (0.959) | 0.8478 | 0.1036-4.4549 |
| 2 | Always | 0 (0%) | 5 (100%) | 0.9844 (0.988) | 0 | 0-Inf | 0.9944 (0.994) | 0 | 0-Inf |
| Household burns wood | |  |  |  |  |  |  |  |  |
| 0 | Never |  |  |  |  |  |  |  |  |
| 1 | Sometimes | 37 (8.4%) | 406 (91.6%) | 0.0183 (0.101) | 3.5314 | 1.3877-11.9379 | 0.459 (0.891) | 0.5819 | 0.1467-2.7316 |
| 2 | Always | 14 (4.1%) | 326 (95.9%) | 0.376 (0.568) | 1.6641 | 0.5858-5.9446 | 0.5142 (0.891) | 0.5828 | 0.117-3.1644 |
| Electricity use | |  |  |  |  |  |  |  |  |
| 0 | Never |  |  |  |  |  |  |  |  |
| 1 | Sometimes | 9 (10.2%) | 79 (89.8%) | 0.0981 (0.278) | 1.9017 | 0.8376-3.9084 | 0.5623 (0.891) | 0.731 | 0.2407-2.0414 |
| 2 | Always | 7 (4.3%) | 157 (95.7%) | 0.4818 (0.669) | 0.7442 | 0.3-1.5959 | 0.7035 (0.891) | 0.7994 | 0.2374-2.4392 |
| Floor |  |  |  |  |  |  |  |  |  |
| 0 | Any flooring |  |  |  |  |  |  |  |  |
| 1 | Mud | 25 (5%) | 480 (95%) | 0.2133 (0.392) | 0.7066 | 0.4061-1.2195 | 0.3544 (0.853) | 0.6852 | 0.3059-1.5273 |
| Maternal Education | | |  |  |  |  |  |  |  |
| 0 | Formal |  |  |  |  |  |  |  |  |
| 1 | Informal | 41 (7.5%) | 505 (92.5%) | 0.0121 (0.075) | 2.2153 | 1.2196-4.2645 | 0.5634 (0.891) | 1.2709 | 0.5744-2.9545 |
| Maternal Occupation | | |  |  |  |  |  |  |  |
| 0 | Professional Employment | |  |  |  |  |  |  |  |
| 1 | Housewife | 35 (7.2%) | 454 (92.8%) | 0.1753 (0.351) | 1.499 | 0.8455-2.7464 | 0.2104 (0.715) | 1.6167 | 0.7716-3.501 |
| 2 | Farming | 2 (2.4%) | 83 (97.6%) | 0.3155 (0.511) | 0.4685 | 0.0736-1.6663 | 0.2423 (0.761) | 3.2412 | 0.3539-20.7134 |
| Mattress | |  |  |  |  |  |  |  |  |
| 0 | Any mattress |  |  |  |  |  |  |  |  |
| 1 | Grass/No mattress | 6 (3.2%) | 181 (96.8%) | 0.0934 (0.276) | 0.4776 | 0.181-1.0489 | 0.0615 (0.516) | 0.3264 | 0.0918-0.9899 |
| Roof |  |  |  |  |  |  |  |  |  |
| 0 | Any roofing except thatched |  |  |  |  |  |  |  |  |
| 1 | Thatched roof | 0 (0%) | 21 (100%) | 0.9863 (0.988) | 0 | 0-Inf | 0.9855 (0.994) | 0 | 0-Inf |
| Wall |  |  |  |  |  |  |  |  |  |
| 0 | Cement/Brick/Iron walls | |  |  |  |  |  |  |  |
| 1 | Wood/Grass | 50 (7.5%) | 614 (92.5%) | 0.0017 (0.021) | 4.4463 | 1.9286-12.8992 | 0.075 (0.516) | 2.8586 | 0.9626-10.071 |
| What the child sleeps on | | |  |  |  |  |  |  |  |
| 0 | Bed |  |  |  |  |  |  |  |  |
| 1 | Floor | 17 (5.2%) | 308 (94.8%) | 0.564 (0.738) | 0.841 | 0.456-1.4907 | 0.2464 (0.761) | 2.0824 | 0.5759-6.9847 |
| **HEALTH FACTORS** | | | | | | | | | |
| Cockroach skin prick test | | |  |  |  |  |  |  |  |
| 0 | Negative |  |  |  |  |  |  |  |  |
| 1 | Positive | 2 (9.1%) | 20 (90.9%) | 0.5145 (0.686) | 1.6358 | 0.2569-5.8079 | 0.5781 (0.891) | 0.4659 | 0.0181-4.4855 |
| Child has asthma | |  |  |  |  |  |  |  |  |
| 0 | No |  |  |  |  |  |  |  |  |
| 1 | Yes | 2 (6.5%) | 29 (93.5%) | 0.8824 (0.963) | 1.1165 | 0.1775-3.8445 | 0.8996 (0.972) | 0.8539 | 0.0429-6.8501 |
| Child has hay fever | |  |  |  |  |  |  |  |  |
| 0 | No |  |  |  |  |  |  |  |  |
| 1 | Yes | 5 (6.9%) | 67 (93.1%) | 0.6776 (0.867) | 1.2239 | 0.4149-2.9013 | 0.4729 (0.891) | 2.0347 | 0.259-13.0435 |
| Child has had hay fever in last year | | |  |  |  |  |  |  |  |
| 0 | No |  |  |  |  |  |  |  |  |
| 1 | Yes | 4 (5%) | 76 (95%) | 0.7384 (0.881) | 0.8369 | 0.2482-2.1186 | 0.26 (0.769) | 0.2682 | 0.0226-2.2989 |
| Child with rash in last year | | |  |  |  |  |  |  |  |
| 0 | No |  |  |  |  |  |  |  |  |
| 1 | Yes | 9 (5.4%) | 159 (94.6%) | 0.7691 (0.886) | 0.8958 | 0.4033-1.7834 | 0.7986 (0.921) | 1.1366 | 0.4026-2.9249 |
| Child has wheeze in last year | | |  |  |  |  |  |  |  |
| 0 | No |  |  |  |  |  |  |  |  |
| 1 | Yes | 1 (1.3%) | 74 (98.7%) | 0.117 (0.284) | 0.2035 | 0.0114-0.9468 | 0.0376 (0.511) | 0.0147 | 0.0001-0.3129 |
| Dust mite skin prick test | | |  |  |  |  |  |  |  |
| 0 | Negative |  |  |  |  |  |  |  |  |
| 1 | Positive | 3 (21.4%) | 11 (78.6%) | 0.0222 (0.108) | 4.5944 | 1.0166-15.253 | 0.2091 (0.715) | 4.7523 | 0.3923-55.0134 |
| Father with Asthma | | |  |  |  |  |  |  |  |
| 0 | No |  |  |  |  |  |  |  |  |
| 1 | Yes | 2 (6.1%) | 31 (93.9%) | 0.9559 (0.988) | 1.042 | 0.1659-3.5724 | 0.1591 (0.715) | 0.0643 | 0.0011-2.3482 |
| Father with Hay Fever | | |  |  |  |  |  |  |  |
| 0 | No |  |  |  |  |  |  |  |  |
| 1 | Yes | 2 (12.5%) | 14 (87.5%) | 0.2658 (0.463) | 2.3531 | 0.3635-8.7146 | 0.3523 (0.853) | 6.4269 | 0.0906-231.6297 |
| Father with wheeze | | |  |  |  |  |  |  |  |
| 0 | No |  |  |  |  |  |  |  |  |
| 1 | Yes | 4 (16.7%) | 20 (83.3%) | 0.0307 (0.123) | 3.4 | 0.9624-9.3936 | 0.0049 (0.204) | 220.2642 | 6.3475-12314.0595 |
| Mother with asthma | | |  |  |  |  |  |  |  |
| 0 | No |  |  |  |  |  |  |  |  |
| 1 | Yes | 2 (4.5%) | 42 (95.5%) | 0.7087 (0.867) | 0.7592 | 0.1217-2.56 | 0.42 (0.891) | 0.396 | 0.0225-2.6993 |
| Mother with hay fever | | |  |  |  |  |  |  |  |
| 0 | No |  |  |  |  |  |  |  |  |
| 1 | Yes | 2 (12.5%) | 14 (87.5%) | 0.2658 (0.463) | 2.3531 | 0.3635-8.7146 | 0.1959 (0.715) | 12.0889 | 0.1636-434.8714 |
| Mother with wheeze | | |  |  |  |  |  |  |  |
| 0 | No |  |  |  |  |  |  |  |  |
| 1 | Yes | 2 (7.1%) | 26 (92.9%) | 0.7655 (0.886) | 1.2496 | 0.198-4.337 | 0.6209 (0.891) | 0.4776 | 0.0144-6.1645 |
| **ENVIRONMENTAL FACTORS** | | | | | | | | | |
| Application of dung to farm fields | | |  |  |  |  |  |  |  |
| 0 | No |  |  |  |  |  |  |  |  |
| 1 | Yes | 3 (17.6%) | 14 (82.4%) | 0.0496 (0.177) | 3.5975 | 0.8112-11.4492 | 0.0881 (0.529) | 5.7885 | 0.7001-42.597 |
| Cigarette smokers in the house | | |  |  |  |  |  |  |  |
| 0 | No |  |  |  |  |  |  |  |  |
| 1 | Yes | 6 (10.3%) | 52 (89.7%) | 0.1377 (0.302) | 1.9662 | 0.7282-4.4781 | 0.4606 (0.891) | 1.6017 | 0.408-5.2063 |
| Cooking area | |  |  |  |  |  |  |  |  |
| 0 | Outside living area | |  |  |  |  |  |  |  |
| 1 | Inside living area | 1 (0.3%) | 360 (99.7%) | 0.0004 (0.006) | 0.0271 | 0.0015-0.1241 | 0.0087 (0.204) | 0.0389 | 0.0015-0.2716 |
| Have a cat | |  |  |  |  |  |  |  |  |
| 0 | No |  |  |  |  |  |  |  |  |
| 1 | Yes | 21 (7.3%) | 267 (92.7%) | 0.209 (0.392) | 1.4342 | 0.8057-2.4979 | 0.7994 (0.921) | 0.8998 | 0.3894-2.0005 |
| Have a cow | |  |  |  |  |  |  |  |  |
| 0 | No |  |  |  |  |  |  |  |  |
| 1 | Yes | 12 (5.9%) | 191 (94.1%) | 0.9602 (0.988) | 1.0169 | 0.504-1.9074 | 0.098 (0.529) | 0.3803 | 0.1139-1.1376 |
| Have a dog | |  |  |  |  |  |  |  |  |
| 0 | No |  |  |  |  |  |  |  |  |
| 1 | Yes | 31 (8%) | 356 (92%) | 0.0193 (0.101) | 1.9266 | 1.1152-3.3669 | 0.0161 (0.275) | 2.6306 | 1.2052-5.8783 |
| Have a hen | |  |  |  |  |  |  |  |  |
| 0 | No |  |  |  |  |  |  |  |  |
| 1 | Yes | 17 (7.8%) | 201 (92.2%) | 0.162 (0.334) | 1.5268 | 0.8244-2.7202 | 0.7072 (0.891) | 0.8517 | 0.3594-1.9358 |
| Have a horse | |  |  |  |  |  |  |  |  |
| 0 | No |  |  |  |  |  |  |  |  |
| 1 | Yes | 9 (9.4%) | 87 (90.6%) | 0.1238 (0.29) | 1.7991 | 0.8012-3.6372 | 0.5155 (0.891) | 0.6866 | 0.2103-2.0734 |
| Have a pig | |  |  |  |  |  |  |  |  |
| 0 | No |  |  |  |  |  |  |  |  |
| 1 | Yes | 1 (20%) | 4 (80%) | 0.2114 (0.392) | 4.088 | 0.2071-28.2251 | 0.749 (0.91) | 0.4648 | 0.0009-26.0042 |
| Have a sheep | |  |  |  |  |  |  |  |  |
| 0 | No |  |  |  |  |  |  |  |  |
| 1 | Yes | 12 (7.6%) | 146 (92.4%) | 0.3042 (0.505) | 1.4164 | 0.6993-2.6698 | 0.2065 (0.715) | 2.0239 | 0.6644-6.0035 |
| Source of water | |  |  |  |  |  |  |  |  |
| 0 | Piped |  |  |  |  |  |  |  |  |
| 1 | Well | 6 (7.6%) | 73 (92.4%) | 0.4364 (0.631) | 1.4205 | 0.5297-3.1996 | 0.5133 (0.891) | 0.6873 | 0.205-1.9988 |
| 2 | River/Stream | 3 (13.6%) | 19 (86.4%) | 0.1165 (0.284) | 2.7288 | 0.6253-8.3793 | 0.0516 (0.516) | 7.0939 | 0.8366-47.8968 |
| Type of toilet | |  |  |  |  |  |  |  |  |
| 0 | Any toilet |  |  |  |  |  |  |  |  |
| 1 | None/Bush/Field | 5 (6.8%) | 68 (93.2%) | 0.7018 (0.867) | 1.2044 | 0.4085-2.8535 | 0.6505 (0.891) | 0.7187 | 0.1528-2.7933 |
| Waste disposal | |  |  |  |  |  |  |  |  |
| 0 | Garbage Bin |  |  |  |  |  |  |  |  |
| 1 | Open Field | 21 (10.8%) | 173 (89.2%) | 0.0062 (0.046) | 2.2476 | 1.244-3.9894 | 0.0618 (0.516) | 2.0484 | 0.961-4.3627 |
| 2 | Pit | 3 (2.1%) | 140 (97.9%) | 0.1309 (0.297) | 0.3968 | 0.0943-1.1314 | 0.107 (0.529) | 0.2669 | 0.039-1.0871 |
| **HEMATOLOGICAL FACTORS** | | | | | | | | | |
| Hematocrit | |  |  |  |  |  |  |  |  |
| Continuous | | - | - | 0.7937 (0.886) | 1.002 | 0.9715-1.0136 | 0.6825 (0.891) | 0.9731 | 0.7843-1.0219 |
| Hemoglobin | |  |  |  |  |  |  |  |  |
| Continuous | | - | - | 0.0525 (0.178) | 1.1541 | 0.9897-1.3289 | 0.6737 (0.891) | 0.8889 | 0.5503-1.5241 |
| Lymphocytes’ count | |  |  |  |  |  |  |  |  |
| 0 | Normal |  |  |  |  |  |  |  |  |
| 1 | Low | 12 (3.2%) | 358 (96.8%) | 0.009 (0.061) | 0.4174 | 0.2076-0.7794 | 0.6225 (0.891) | 0.8069 | 0.3344-1.8713 |
| 2 | High | 1 (14.3%) | 6 (85.7%) | 0.5037 (0.685) | 2.0754 | 0.1086-12.5419 | 0.9165 (0.972) | 0.8576 | 0.0283-12.2435 |
| Mean Corpuscular Hemoglobin | | |  |  |  |  |  |  |  |
| Continuous | | - | - | 0.7144 (0.867) | 0.9752 | 0.8582-1.12 | 0.3438 (0.853) | 1.2355 | 0.8239-1.8629 |
| Mean Corpuscular Hemoglobin Concentration | | | |  |  |  |  |  |  |
| Continuous | | - | - | 0.1421 (0.302) | 1.0651 | 0.9662-1.1774 | 0.5465 (0.891) | 1.0322 | 0.9065-1.1475 |
| Mean Corpuscular Volume | | |  |  |  |  |  |  |  |
| Continuous | | - | - | 0.4155 (0.614) | 0.9848 | 0.9528-1.0269 | 0.9289 (0.972) | 1.0055 | 0.9329-1.1777 |
| Platelet count | |  |  |  |  |  |  |  |  |
| Continuous | | - | - | 0.373 (0.568) | 1.0014 | 0.9983-1.0044 | 0.7663 (0.914) | 1.0007 | 0.9962-1.0052 |
| Red Blood Cell count | | |  |  |  |  |  |  |  |
| Continuous | | - | - | 0.025 (0.113) | 1.6685 | 1.0436-2.5796 | 0.5641 (0.891) | 1.804 | 0.2447-12.7374 |
| White Blood Cell count | | |  |  |  |  |  |  |  |
| Continuous | | - | - | 0.003 (0.027) | 0.8172 | 0.7112-0.9287 | 0.3026 (0.853) | 0.9038 | 0.7389-1.0872 |
