# Supplementary material for: Machine learning-based risk factor analysis and prevalence prediction of intestinal parasitic infections using epidemiological survey data
Source: PLoS Negl Trop Dis. 2022 Jun 14;16(6):e0010517. doi: 10.1371/journal.pntd.0010517 (PMC9236253; doi:10.1371/journal.pntd.0010517)
Supplement: S3 Table — For each risk factor, corresponding references and survey results are provided. Adjusted p-values are provided in parenthesis. (DOCX) [file pntd.0010517.s005.docx]

**S3 Table.** Univariate and multivariate logistic regression analysis of risk factors for infection with any helminth. For each risk factor, corresponding references and survey results are provided. Adjusted p-values are provided in parenthesis.

| **Variable Name** | **Meaning** | **Helminth (+)** | **Helminth (-)** | **P value (uni)** | **COR** | **CI-95% (uni)** | **P value (multi)** | **AOR** | **CI-95% (multi)** |
| --- | --- | --- | --- | --- | --- | --- | --- | --- | --- |
| **DEMOGRAPHIC FACTORS** | | | | | | | | | |
| Age |  |  |  |  |  |  |  |  |  |
| 0 | >10 |  |  |  |  |  |  |  |  |
| 1 | 6 to 10 | 57 (14%) | 351 (86%) | 0.3836 (0.982) | 0.8458 | 0.5787-1.2312 | 0.4076 (0.854) | 0.8289 | 0.5304-1.2918 |
| 2 | <6 | 13 (14.9%) | 74 (85.1%) | 0.7859 (0.982) | 0.915 | 0.4637-1.6879 | 0.5666 (0.91) | 1.2524 | 0.5631-2.647 |
| Deworming | |  |  |  |  |  |  |  |  |
| 0 | Not dewormed | |  |  |  |  |  |  |  |
| 1 | Dewormed | 109 (14.5%) | 643 (85.5%) | 0.3233 (0.982) | 0.8065 | 0.5314-1.2507 | 0.5478 (0.91) | 0.8623 | 0.5372-1.4156 |
| Family Size | |  |  |  |  |  |  |  |  |
| 0 | <6 |  |  |  |  |  |  |  |  |
| 1 | 6 to 9 | 35 (11.6%) | 267 (88.4%) | 0.0459 (0.559) | 0.658 | 0.4312-0.9832 | 0.0605 (0.587) | 0.6394 | 0.3963-1.0108 |
| 2 | >9 | 4 (20%) | 16 (80%) | 0.6901 (0.982) | 1.2549 | 0.354-3.5039 | 0.3904 (0.854) | 1.7045 | 0.4431-5.3351 |
| Residence | |  |  |  |  |  |  |  |  |
| 0 | Rural |  |  |  |  |  |  |  |  |
| 1 | Urban | 70 (13.3%) | 456 (86.7%) | 0.0892 (0.758) | 0.7334 | 0.5125-1.049 | 0.1317 (0.738) | 0.6699 | 0.3948-1.1213 |
| Sex |  |  |  |  |  |  |  |  |  |
| 0 | Male |  |  |  |  |  |  |  |  |
| 1 | Female | 73 (14.1%) | 445 (85.9%) | 0.3523 (0.982) | 0.844 | 0.5902-1.2078 | 0.2295 (0.78) | 0.7844 | 0.5273-1.166 |
| **SOCIOECONOMIC FACTORS** | | | | | | | | | |
| Bed |  |  |  |  |  |  |  |  |  |
| 0 | No |  |  |  |  |  |  |  |  |
| 1 | Yes | 49 (14.9%) | 279 (85.1%) | 0.9324 (0.982) | 0.9839 | 0.6721-1.4257 | 0.6886 (0.956) | 1.1615 | 0.5571-2.4134 |
| Household burns charcoal | | |  |  |  |  |  |  |  |
| 0 | Never |  |  |  |  |  |  |  |  |
| 1 | Sometimes | 56 (16%) | 293 (84%) | 0.3561 (0.982) | 1.3697 | 0.7241-2.786 | 0.6738 (0.956) | 1.1961 | 0.5303-2.8368 |
| 2 | Always | 74 (14.9%) | 421 (85.1%) | 0.488 (0.982) | 1.2597 | 0.6782-2.5286 | 0.5887 (0.91) | 1.2517 | 0.5694-2.925 |
| Household burns dung | |  |  |  |  |  |  |  |  |
| 0 | Never |  |  |  |  |  |  |  |  |
| 1 | Sometimes | 40 (15.9%) | 212 (84.1%) | 0.6907 (0.982) | 1.0849 | 0.72-1.61 | 0.9334 (0.995) | 0.9779 | 0.576-1.6466 |
| 2 | Always | 6 (14.3%) | 36 (85.7%) | 0.9254 (0.982) | 0.9583 | 0.3555-2.1791 | 0.5845 (0.91) | 1.315 | 0.453-3.3218 |
| Household burns gas | |  |  |  |  |  |  |  |  |
| 0 | Never |  |  |  |  |  |  |  |  |
| 1 | Sometimes | 24 (24%) | 76 (76%) | 0.0084 (0.324) | 1.9574 | 1.1684-3.1829 | 0.1189 (0.738) | 1.6057 | 0.8736-2.8849 |
| 2 | Always | 2 (28.6%) | 5 (71.4%) | 0.2812 (0.982) | 2.4793 | 0.3522-11.6505 | 0.7536 (0.995) | 1.3817 | 0.1345-8.9768 |
| Household burns leaves | |  |  |  |  |  |  |  |  |
| 0 | Never |  |  |  |  |  |  |  |  |
| 1 | Sometimes | 51 (15.5%) | 277 (84.5%) | 0.8032 (0.982) | 1.049 | 0.7164-1.5232 | 0.2104 (0.778) | 0.7027 | 0.4038-1.2209 |
| 2 | Always | 5 (13.2%) | 33 (86.8%) | 0.766 (0.982) | 0.8633 | 0.2894-2.089 | 0.2874 (0.854) | 0.5514 | 0.1653-1.5356 |
| Household burns nafta | |  |  |  |  |  |  |  |  |
| 0 | Never |  |  |  |  |  |  |  |  |
| 1 | Sometimes | 10 (31.2%) | 22 (68.8%) | 0.0112 (0.324) | 2.7098 | 1.203-5.7115 | 0.0989 (0.738) | 2.1711 | 0.8407-5.377 |
| 2 | Always | 2 (40%) | 3 (60%) | 0.1327 (0.809) | 3.9744 | 0.5198-24.2049 | 0.0067 (0.208) | 29.7464 | 2.3166-379.5405 |
| Household burns wood | |  |  |  |  |  |  |  |  |
| 0 | Never |  |  |  |  |  |  |  |  |
| 1 | Sometimes | 71 (16%) | 372 (84%) | 0.5124 (0.982) | 1.1885 | 0.7194-2.0313 | 0.8829 (0.995) | 1.0552 | 0.5242-2.2022 |
| 2 | Always | 49 (14.4%) | 291 (85.6%) | 0.8639 (0.982) | 1.0486 | 0.6164-1.8328 | 0.5883 (0.91) | 1.2177 | 0.6043-2.5287 |
| Electricity use | |  |  |  |  |  |  |  |  |
| 0 | Never |  |  |  |  |  |  |  |  |
| 1 | Sometimes | 15 (17%) | 73 (83%) | 0.655 (0.982) | 1.1448 | 0.6118-2.0196 | 0.9195 (0.995) | 0.9641 | 0.4616-1.9196 |
| 2 | Always | 22 (13.4%) | 142 (86.6%) | 0.56 (0.982) | 0.8632 | 0.515-1.3914 | 0.633 (0.956) | 0.8478 | 0.4234-1.6474 |
| Floor |  |  |  |  |  |  |  |  |  |
| 0 | Any flooring |  |  |  |  |  |  |  |  |
| 1 | Mud | 72 (14.3%) | 433 (85.7%) | 0.4515 (0.982) | 0.8718 | 0.6097-1.247 | 0.1509 (0.738) | 0.7174 | 0.4553-1.1286 |
| Maternal Education | | |  |  |  |  |  |  |  |
| 0 | Formal |  |  |  |  |  |  |  |  |
| 1 | Informal | 90 (16.5%) | 456 (83.5%) | 0.1566 (0.809) | 1.3057 | 0.9061-1.898 | 0.2905 (0.854) | 1.2739 | 0.8152-2.0038 |
| Maternal Occupation | | |  |  |  |  |  |  |  |
| 0 | Professional Employment | |  |  |  |  |  |  |  |
| 1 | Housewife | 65 (13.3%) | 424 (86.7%) | 0.0493 (0.559) | 0.6887 | 0.4744-0.9992 | 0.1934 (0.778) | 0.7564 | 0.4961-1.1525 |
| 2 | Farming | 10 (11.8%) | 75 (88.2%) | 0.1577 (0.809) | 0.599 | 0.2786-1.1713 | 0.0461 (0.522) | 0.3961 | 0.1499-0.9393 |
| Mattress | |  |  |  |  |  |  |  |  |
| 0 | Any mattress |  |  |  |  |  |  |  |  |
| 1 | Grass/No mattress | 39 (20.9%) | 148 (79.1%) | 0.0143 (0.324) | 1.6681 | 1.0981-2.4952 | 0.0054 (0.208) | 2.1136 | 1.2421-3.5727 |
| Roof |  |  |  |  |  |  |  |  |  |
| 0 | Any roofing except thatched |  |  |  |  |  |  |  |  |
| 1 | Thatched roof | 1 (4.8%) | 20 (95.2%) | 0.2115 (0.899) | 0.2766 | 0.0154-1.343 | 0.0782 (0.665) | 0.104 | 0.004-0.8404 |
| Wall |  |  |  |  |  |  |  |  |  |
| 0 | Cement/Brick/Iron walls | |  |  |  |  |  |  |  |
| 1 | Wood/Grass | 99 (14.9%) | 565 (85.1%) | 0.8272 (0.982) | 0.9576 | 0.653-1.4239 | 0.2174 (0.778) | 0.7321 | 0.4466-1.2057 |
| What the child sleeps on | | |  |  |  |  |  |  |  |
| 0 | Bed |  |  |  |  |  |  |  |  |
| 1 | Floor | 48 (14.8%) | 277 (85.2%) | 0.8494 (0.982) | 0.9641 | 0.6573-1.399 | 0.3734 (0.854) | 0.7217 | 0.3504-1.4759 |
| **HEALTH FACTORS** | | | | | | | | | |
| Cockroach skin prick test | | |  |  |  |  |  |  |  |
| 0 | Negative |  |  |  |  |  |  |  |  |
| 1 | Positive | 4 (18.2%) | 18 (81.8%) | 0.6808 (0.982) | 1.2593 | 0.3597-3.4353 | 0.8904 (0.995) | 0.9115 | 0.2129-3.1138 |
| Child has asthma | |  |  |  |  |  |  |  |  |
| 0 | No |  |  |  |  |  |  |  |  |
| 1 | Yes | 4 (12.9%) | 27 (87.1%) | 0.7315 (0.982) | 0.8298 | 0.2425-2.1631 | 0.3596 (0.854) | 0.5664 | 0.1443-1.722 |
| Child has hay fever | |  |  |  |  |  |  |  |  |
| 0 | No |  |  |  |  |  |  |  |  |
| 1 | Yes | 10 (13.9%) | 62 (86.1%) | 0.77 (0.982) | 0.9018 | 0.4257-1.7275 | 0.4716 (0.908) | 1.597 | 0.4358-5.6487 |
| Child has had hay fever in last year | | |  |  |  |  |  |  |  |
| 0 | No |  |  |  |  |  |  |  |  |
| 1 | Yes | 10 (12.5%) | 70 (87.5%) | 0.502 (0.982) | 0.79 | 0.3745-1.5041 | 0.1469 (0.738) | 0.3834 | 0.0982-1.3207 |
| Child with rash in last year | | |  |  |  |  |  |  |  |
| 0 | No |  |  |  |  |  |  |  |  |
| 1 | Yes | 25 (14.9%) | 143 (85.1%) | 0.9384 (0.982) | 0.9817 | 0.6034-1.5447 | 0.7783 (0.995) | 1.0778 | 0.6293-1.7937 |
| Child has had wheeze in last year | | |  |  |  |  |  |  |  |
| 0 | No |  |  |  |  |  |  |  |  |
| 1 | Yes | 13 (17.3%) | 62 (82.7%) | 0.5692 (0.982) | 1.1995 | 0.6157-2.1768 | 0.5399 (0.91) | 1.2682 | 0.5725-2.6413 |
| Dust mite skin prick test | | |  |  |  |  |  |  |  |
| 0 | Negative |  |  |  |  |  |  |  |  |
| 1 | Positive | 4 (28.6%) | 10 (71.4%) | 0.1665 (0.809) | 2.2899 | 0.6212-6.9521 | 0.152 (0.738) | 2.7718 | 0.6244-10.8107 |
| Father with Asthma | | |  |  |  |  |  |  |  |
| 0 | No |  |  |  |  |  |  |  |  |
| 1 | Yes | 6 (18.2%) | 27 (81.8%) | 0.6123 (0.982) | 1.2631 | 0.4644-2.9202 | 0.5198 (0.91) | 1.5098 | 0.3849-4.9037 |
| Father with Hay Fever | | |  |  |  |  |  |  |  |
| 0 | No |  |  |  |  |  |  |  |  |
| 1 | Yes | 5 (31.2%) | 11 (68.8%) | 0.0787 (0.758) | 2.6178 | 0.8144-7.3177 | 0.0386 (0.522) | 4.5583 | 1.0279-19.2238 |
| Father with wheeze | | |  |  |  |  |  |  |  |
| 0 | No |  |  |  |  |  |  |  |  |
| 1 | Yes | 5 (20.8%) | 19 (79.2%) | 0.4274 (0.982) | 1.5002 | 0.4909-3.8025 | 0.921 (0.995) | 0.9245 | 0.1831-4.1919 |
| Mother with asthma | | |  |  |  |  |  |  |  |
| 0 | No |  |  |  |  |  |  |  |  |
| 1 | Yes | 7 (15.9%) | 37 (84.1%) | 0.8741 (0.982) | 1.0693 | 0.4293-2.3074 | 0.8064 (0.995) | 1.1413 | 0.369-3.1291 |
| Mother with hay fever | | |  |  |  |  |  |  |  |
| 0 | No |  |  |  |  |  |  |  |  |
| 1 | Yes | 2 (12.5%) | 14 (87.5%) | 0.772 (0.982) | 0.802 | 0.1251-2.9117 | 0.3591 (0.854) | 0.3798 | 0.0349-2.3626 |
| Mother with wheeze | | |  |  |  |  |  |  |  |
| 0 | No |  |  |  |  |  |  |  |  |
| 1 | Yes | 5 (17.9%) | 23 (82.1%) | 0.6766 (0.982) | 1.2329 | 0.4086-3.0512 | 0.337 (0.854) | 1.8668 | 0.476-6.3384 |
| **ENVIRONMENTAL FACTORS** | | | | | | | | | |
| Application of dung to farm fields | | |  |  |  |  |  |  |  |
| 0 | No |  |  |  |  |  |  |  |  |
| 1 | Yes | 3 (17.6%) | 14 (82.4%) | 0.7651 (0.982) | 1.2117 | 0.2767-3.7711 | 0.9073 (0.995) | 0.9178 | 0.1794-3.4916 |
| Cigarette smokers in the house | | |  |  |  |  |  |  |  |
| 0 | No |  |  |  |  |  |  |  |  |
| 1 | Yes | 9 (15.5%) | 49 (84.5%) | 0.9225 (0.982) | 1.0371 | 0.467-2.0633 | 0.9069 (0.995) | 0.9522 | 0.3945-2.0703 |
| Cooking area | |  |  |  |  |  |  |  |  |
| 0 | Outside living area | |  |  |  |  |  |  |  |
| 1 | Inside living area | 46 (12.7%) | 315 (87.3%) | 0.1158 (0.809) | 0.7378 | 0.5015-1.072 | 0.7971 (0.995) | 0.9279 | 0.5227-1.6402 |
| Have a cat | |  |  |  |  |  |  |  |  |
| 0 | No |  |  |  |  |  |  |  |  |
| 1 | Yes | 41 (14.2%) | 247 (85.8%) | 0.6334 (0.982) | 0.9088 | 0.6084-1.3368 | 0.5666 (0.91) | 0.8686 | 0.5315-1.3961 |
| Have a cow | |  |  |  |  |  |  |  |  |
| 0 | No |  |  |  |  |  |  |  |  |
| 1 | Yes | 27 (13.3%) | 176 (86.7%) | 0.4257 (0.982) | 0.8324 | 0.5211-1.2893 | 0.6888 (0.956) | 0.8844 | 0.4762-1.5904 |
| Have a dog | |  |  |  |  |  |  |  |  |
| 0 | No |  |  |  |  |  |  |  |  |
| 1 | Yes | 56 (14.5%) | 331 (85.5%) | 0.6653 (0.982) | 0.9226 | 0.6381-1.3252 | 0.7989 (0.995) | 1.0601 | 0.6731-1.6565 |
| Have a hen | |  |  |  |  |  |  |  |  |
| 0 | No |  |  |  |  |  |  |  |  |
| 1 | Yes | 33 (15.1%) | 185 (84.9%) | 0.9762 (0.994) | 1.0064 | 0.6514-1.5206 | 0.9516 (0.995) | 1.0161 | 0.6002-1.6831 |
| Have a horse | |  |  |  |  |  |  |  |  |
| 0 | No |  |  |  |  |  |  |  |  |
| 1 | Yes | 13 (13.5%) | 83 (86.5%) | 0.6581 (0.982) | 0.8706 | 0.4515-1.5565 | 0.9052 (0.995) | 0.9545 | 0.4292-2.002 |
| Have a pig | |  |  |  |  |  |  |  |  |
| 0 | No |  |  |  |  |  |  |  |  |
| 1 | Yes | 0 (0%) | 5 (100%) | 0.983 (0.994) | 0 | 0-Inf | 0.9814 (0.995) | 0 | 0-Inf |
| Have a sheep | |  |  |  |  |  |  |  |  |
| 0 | No |  |  |  |  |  |  |  |  |
| 1 | Yes | 23 (14.6%) | 135 (85.4%) | 0.8421 (0.982) | 0.9521 | 0.5751-1.5181 | 0.995 (0.995) | 0.998 | 0.53-1.816 |
| Source of water | |  |  |  |  |  |  |  |  |
| 0 | Piped |  |  |  |  |  |  |  |  |
| 1 | Well | 16 (20.3%) | 63 (79.7%) | 0.1948 (0.883) | 1.4685 | 0.7972-2.5659 | 0.2128 (0.778) | 1.5678 | 0.7562-3.1331 |
| 2 | River/Stream | 2 (9.1%) | 20 (90.9%) | 0.4639 (0.982) | 0.5782 | 0.0916-2.0139 | 0.3936 (0.854) | 0.4951 | 0.0707-2.0724 |
| Type of toilet | |  |  |  |  |  |  |  |  |
| 0 | Any toilet |  |  |  |  |  |  |  |  |
| 1 | None/Bush/Field | 12 (16.4%) | 61 (83.6%) | 0.7346 (0.982) | 1.1183 | 0.5601-2.0633 | 0.4146 (0.854) | 0.7175 | 0.3103-1.545 |
| Waste disposal | |  |  |  |  |  |  |  |  |
| 0 | Garbage Bin |  |  |  |  |  |  |  |  |
| 1 | Open Field | 32 (16.5%) | 162 (83.5%) | 0.5466 (0.982) | 1.1452 | 0.7284-1.7636 | 0.6722 (0.956) | 1.1236 | 0.6482-1.9128 |
| 2 | Pit | 21 (14.7%) | 122 (85.3%) | 0.9938 (0.994) | 0.998 | 0.5835-1.6416 | 0.9702 (0.995) | 1.0111 | 0.5535-1.7823 |
| **HEMATOLOGICAL FACTORS** | | | | | | | | | |
| Hematocrit | |  |  |  |  |  |  |  |  |
| Continuous | | - | - | 0.1473 (0.809) | 1.0219 | 1.0021-1.0534 | 0.0092 (0.208) | 1.0764 | 1.0253-1.1513 |
| Hemoglobin | |  |  |  |  |  |  |  |  |
| Continuous | | - | - | 0.6864 (0.982) | 0.9784 | 0.8818-1.089 | 0.4808 (0.908) | 0.9143 | 0.7209-1.1957 |
| Lymphocytes’ count | | |  |  |  |  |  |  |  |
| 0 |  |  |  |  |  |  |  |  |  |
| 1 | Low | 56 (15.1%) | 314 (84.9%) | 0.9106 (0.982) | 1.0212 | 0.705-1.4701 | 0.399 (0.854) | 1.2012 | 0.783-1.8388 |
| 2 | High | 2 (28.6%) | 5 (71.4%) | 0.3267 (0.982) | 2.2905 | 0.3244-10.818 | 0.4752 (0.908) | 1.956 | 0.2403-11.2039 |
| Mean Corpuscular Hemoglobin | | |  |  |  |  |  |  |  |
| Continuous | | - | - | 0.036 (0.559) | 0.9122 | 0.8374-0.9952 | 0.0196 (0.333) | 0.8024 | 0.6552-0.9541 |
| Mean Corpuscular Hemoglobin Concentration | | | |  |  |  |  |  |  |
| Continuous | | - | - | 0.4623 (0.982) | 1.0279 | 0.9502-1.1115 | 0.1905 (0.778) | 1.0569 | 0.9765-1.1741 |
| Mean Corpuscular Volume | | |  |  |  |  |  |  |  |
| Continuous | | - | - | 0.4791 (0.982) | 0.9905 | 0.9655-1.0188 | 0.3528 (0.854) | 1.0343 | 0.9784-1.1201 |
| Platelet count | |  |  |  |  |  |  |  |  |
| Continuous | | - | - | 0.8513 (0.982) | 0.9998 | 0.9977-1.0018 | 0.844 (0.995) | 0.9998 | 0.9973-1.0022 |
| Red Blood Cell count | | |  |  |  |  |  |  |  |
| Continuous | | - | - | 0.7016 (0.982) | 1.066 | 0.7718-1.4802 | 0.2628 (0.851) | 0.6418 | 0.2829-1.3931 |
| White Blood Cell count | | |  |  |  |  |  |  |  |
| Continuous | | - | - | 0.6897 (0.982) | 0.9858 | 0.917-1.0557 | 0.9581 (0.995) | 1.0022 | 0.9225-1.0849 |
