# Supplementary material for: Machine learning-based risk factor analysis and prevalence prediction of intestinal parasitic infections using epidemiological survey data
Source: PLoS Negl Trop Dis. 2022 Jun 14;16(6):e0010517. doi: 10.1371/journal.pntd.0010517 (PMC9236253; doi:10.1371/journal.pntd.0010517)
Supplement: S4 Table — For each risk factor, corresponding references and survey results are provided. Adjusted p-values are provided in parenthesis. (DOCX) [file pntd.0010517.s006.docx]

**S4 Table.** Univariate and multivariate logistic regression analysis of risk factors for infection with parasites. For each risk factor, corresponding references and survey results are provided. Adjusted p-values are provided in parenthesis.

| **Variable Name** | **Meaning** | **Parasite (+)** | **Parasite (-)** | **P value (uni)** | **COR** | **CI-95% (uni)** | **P value (multi)** | **AOR** | **CI-95% (multi)** |
| --- | --- | --- | --- | --- | --- | --- | --- | --- | --- |
| **DEMOGRAPHIC FACTORS** | | | | | | | | | |
| Age |  |  |  |  |  |  |  |  |  |
| 0 | >10 |  |  |  |  |  |  |  |  |
| 1 | 6 to 10 | 75 (18.4%) | 333 (81.6%) | 0.1493 (0.441) | 0.7815 | 0.5579-1.0912 | 0.4423 (0.91) | 0.8558 | 0.5745-1.2729 |
| 2 | <6 | 14 (16.1%) | 73 (83.9%) | 0.1933 (0.491) | 0.6655 | 0.3475-1.1958 | 0.8167 (0.91) | 1.0905 | 0.5093-2.2239 |
| Deworming | |  |  |  |  |  |  |  |  |
| 0 | Not dewormed | |  |  |  |  |  |  |  |
| 1 | Dewormed | 150 (19.9%) | 602 (80.1%) | 0.8586 (0.927) | 0.9647 | 0.6553-1.4463 | 0.8997 (0.956) | 1.0293 | 0.6625-1.6295 |
| Family Size | |  |  |  |  |  |  |  |  |
| 0 | <6 |  |  |  |  |  |  |  |  |
| 1 | 6 to 9 | 47 (15.6%) | 255 (84.4%) | 0.0178 (0.134) | 0.6438 | 0.4437-0.9205 | 0.0312 (0.53) | 0.6319 | 0.4131-0.9534 |
| 2 | >9 | 4 (20%) | 16 (80%) | 0.8111 (0.89) | 0.8732 | 0.2471-2.4266 | 0.7449 (0.91) | 0.8191 | 0.2152-2.5155 |
| Residence | |  |  |  |  |  |  |  |  |
| 0 | Rural |  |  |  |  |  |  |  |  |
| 1 | Urban | 84 (16%) | 442 (84%) | 0.0005 (0.008) | 0.5629 | 0.4074-0.7756 | 0.2273 (0.773) | 0.7599 | 0.4847-1.1836 |
| Sex |  |  |  |  |  |  |  |  |  |
| 0 | Male |  |  |  |  |  |  |  |  |
| 1 | Female | 102 (19.7%) | 416 (80.3%) | 0.7523 (0.868) | 0.9498 | 0.6899-1.3096 | 0.5594 (0.91) | 0.8998 | 0.6313-1.2837 |
| **SOCIOECONOMIC FACTORS** | | | | | | | | | |
| Bed |  |  |  |  |  |  |  |  |  |
| 0 | No |  |  |  |  |  |  |  |  |
| 1 | Yes | 63 (19.2%) | 265 (80.8%) | 0.6315 (0.806) | 0.9208 | 0.6542-1.2858 | 0.973 (0.988) | 0.9885 | 0.5043-1.9311 |
| Household burns charcoal | | |  |  |  |  |  |  |  |
| 0 | Never |  |  |  |  |  |  |  |  |
| 1 | Sometimes | 83 (23.8%) | 266 (76.2%) | 0.0274 (0.155) | 2.0402 | 1.116-4.0005 | 0.6998 (0.91) | 1.1662 | 0.5441-2.6203 |
| 2 | Always | 93 (18.8%) | 402 (81.2%) | 0.1949 (0.491) | 1.5126 | 0.8347-2.9459 | 0.6737 (0.91) | 1.1775 | 0.5635-2.6018 |
| Household burns dung | |  |  |  |  |  |  |  |  |
| 0 | Never |  |  |  |  |  |  |  |  |
| 1 | Sometimes | 62 (24.6%) | 190 (75.4%) | 0.0366 (0.178) | 1.4506 | 1.0194-2.0503 | 0.6208 (0.91) | 0.8928 | 0.5675-1.3957 |
| 2 | Always | 8 (19%) | 34 (81%) | 0.9118 (0.963) | 1.046 | 0.4408-2.2093 | 0.4692 (0.91) | 1.3928 | 0.5357-3.2859 |
| Household burns gas | |  |  |  |  |  |  |  |  |
| 0 | Never |  |  |  |  |  |  |  |  |
| 1 | Sometimes | 35 (35%) | 65 (65%) | 0.0001 (0.004) | 2.4195 | 1.5353-3.7625 | 0.0952 (0.607) | 1.5618 | 0.9184-2.624 |
| 2 | Always | 2 (28.6%) | 5 (71.4%) | 0.4859 (0.768) | 1.7974 | 0.2557-8.4247 | 0.7764 (0.91) | 1.3389 | 0.1275-8.5989 |
| Household burns leaves | |  |  |  |  |  |  |  |  |
| 0 | Never |  |  |  |  |  |  |  |  |
| 1 | Sometimes | 86 (26.2%) | 242 (73.8%) | 0.0005 (0.008) | 1.7993 | 1.2926-2.5033 | 0.6402 (0.91) | 1.1203 | 0.6965-1.8086 |
| 2 | Always | 8 (21.1%) | 30 (78.9%) | 0.4677 (0.768) | 1.3502 | 0.5623-2.9042 | 0.6323 (0.91) | 0.7942 | 0.2917-1.9612 |
| Household burns nafta | |  |  |  |  |  |  |  |  |
| 0 | Never |  |  |  |  |  |  |  |  |
| 1 | Sometimes | 14 (43.8%) | 18 (56.2%) | 0.0011 (0.016) | 3.2909 | 1.5794-6.7262 | 0.0671 (0.607) | 2.2002 | 0.9357-5.1161 |
| 2 | Always | 2 (40%) | 3 (60%) | 0.258 (0.566) | 2.8208 | 0.3694-17.1477 | 0.0671 (0.607) | 9.6557 | 0.7609-121.6933 |
| Household burns wood | |  |  |  |  |  |  |  |  |
| 0 | Never |  |  |  |  |  |  |  |  |
| 1 | Sometimes | 105 (23.7%) | 338 (76.3%) | 0.0245 (0.151) | 1.7474 | 1.0906-2.8953 | 0.7104 (0.91) | 1.133 | 0.5937-2.2293 |
| 2 | Always | 60 (17.6%) | 280 (82.4%) | 0.4781 (0.768) | 1.2054 | 0.7276-2.0503 | 0.5438 (0.91) | 1.2286 | 0.6389-2.4243 |
| Electricity use | |  |  |  |  |  |  |  |  |
| 0 | Never |  |  |  |  |  |  |  |  |
| 1 | Sometimes | 24 (27.3%) | 64 (72.7%) | 0.1237 (0.4) | 1.4865 | 0.8831-2.4329 | 0.9572 (0.988) | 0.9836 | 0.5296-1.7832 |
| 2 | Always | 26 (15.9%) | 138 (84.1%) | 0.2121 (0.514) | 0.7468 | 0.4638-1.1642 | 0.4068 (0.91) | 0.773 | 0.4149-1.4048 |
| Floor |  |  |  |  |  |  |  |  |  |
| 0 | Any flooring |  |  |  |  |  |  |  |  |
| 1 | Mud | 96 (19%) | 409 (81%) | 0.3855 (0.723) | 0.8682 | 0.6308-1.1952 | 0.0997 (0.607) | 0.7115 | 0.4738-1.0667 |
| Maternal Education | | |  |  |  |  |  |  |  |
| 0 | Formal |  |  |  |  |  |  |  |  |
| 1 | Informal | 126 (23.1%) | 420 (76.9%) | 0.007 (0.073) | 1.5857 | 1.1386-2.2266 | 0.2022 (0.773) | 1.3007 | 0.8702-1.9545 |
| Maternal Occupation | | |  |  |  |  |  |  |  |
| 0 | Professional Employment | |  |  |  |  |  |  |  |
| 1 | Housewife | 94 (19.2%) | 395 (80.8%) | 0.2728 (0.58) | 0.83 | 0.5951-1.1592 | 0.6807 (0.91) | 0.9233 | 0.6317-1.3519 |
| 2 | Farming | 13 (15.3%) | 72 (84.7%) | 0.1565 (0.443) | 0.6297 | 0.3195-1.1595 | 0.4789 (0.91) | 0.747 | 0.3198-1.6246 |
| Mattress | |  |  |  |  |  |  |  |  |
| 0 | Any mattress |  |  |  |  |  |  |  |  |
| 1 | Grass/No mattress | 44 (23.5%) | 143 (76.5%) | 0.187 (0.491) | 1.2944 | 0.8753-1.8872 | 0.1987 (0.773) | 1.3765 | 0.8409-2.2324 |
| Roof |  |  |  |  |  |  |  |  |  |
| 0 | Any roofing except thatched |  |  |  |  |  |  |  |  |
| 1 | Thatched roof | 1 (4.8%) | 20 (95.2%) | 0.1117 (0.4) | 0.1949 | 0.0108-0.9441 | 0.0796 (0.607) | 0.1291 | 0.006-0.8362 |
| Wall |  |  |  |  |  |  |  |  |  |
| 0 | Cement/Brick/Iron walls | |  |  |  |  |  |  |  |
| 1 | Wood/Grass | 142 (21.4%) | 522 (78.6%) | 0.1182 (0.4) | 1.337 | 0.9348-1.9401 | 0.6187 (0.91) | 0.8903 | 0.5645-1.4125 |
| What the child sleeps on | | |  |  |  |  |  |  |  |
| 0 | Bed |  |  |  |  |  |  |  |  |
| 1 | Floor | 65 (20%) | 260 (80%) | 0.9717 (0.986) | 0.994 | 0.7077-1.3861 | 0.7406 (0.91) | 0.8948 | 0.4622-1.7269 |
| **HEALTH FACTORS** | | | | | | | | | |
| Cockroach skin prick test | | |  |  |  |  |  |  |  |
| 0 | Negative |  |  |  |  |  |  |  |  |
| 1 | Positive | 6 (27.3%) | 16 (72.7%) | 0.3961 (0.723) | 1.5102 | 0.5357-3.7292 | 0.6384 (0.91) | 0.7459 | 0.1991-2.3729 |
| Child has asthma | |  |  |  |  |  |  |  |  |
| 0 | No |  |  |  |  |  |  |  |  |
| 1 | Yes | 6 (19.4%) | 25 (80.6%) | 0.9202 (0.963) | 0.9548 | 0.3505-2.2157 | 0.6846 (0.91) | 0.8069 | 0.2629-2.1431 |
| Child has hay fever | |  |  |  |  |  |  |  |  |
| 0 | No |  |  |  |  |  |  |  |  |
| 1 | Yes | 13 (18.1%) | 59 (81.9%) | 0.6582 (0.814) | 0.8688 | 0.4474-1.5704 | 0.5146 (0.91) | 1.4734 | 0.4502-4.7081 |
| Child has had hay fever in last year | | |  |  |  |  |  |  |  |
| 0 | No |  |  |  |  |  |  |  |  |
| 1 | Yes | 13 (16.2%) | 67 (83.8%) | 0.3746 (0.723) | 0.7563 | 0.3914-1.3569 | 0.0914 (0.607) | 0.3598 | 0.1037-1.1273 |
| Child with rash in last year | | |  |  |  |  |  |  |  |
| 0 | No |  |  |  |  |  |  |  |  |
| 1 | Yes | 35 (20.8%) | 133 (79.2%) | 0.7835 (0.888) | 1.0595 | 0.6936-1.5847 | 0.321 (0.91) | 1.2681 | 0.7858-2.013 |
| Child has wheeze in last year | | |  |  |  |  |  |  |  |
| 0 | No |  |  |  |  |  |  |  |  |
| 1 | Yes | 14 (18.7%) | 61 (81.3%) | 0.7529 (0.868) | 0.9075 | 0.478-1.6141 | 0.3778 (0.91) | 0.7181 | 0.3311-1.4557 |
| Dust mite skin prick test | | |  |  |  |  |  |  |  |
| 0 | Negative |  |  |  |  |  |  |  |  |
| 1 | Positive | 7 (50%) | 7 (50%) | 0.0091 (0.077) | 4.0989 | 1.3872-12.113 | 0.0198 (0.53) | 4.7114 | 1.2708-17.9714 |
| Father with Asthma | | |  |  |  |  |  |  |  |
| 0 | No |  |  |  |  |  |  |  |  |
| 1 | Yes | 8 (24.2%) | 25 (75.8%) | 0.5427 (0.802) | 1.2871 | 0.5355-2.7817 | 0.9666 (0.988) | 0.9742 | 0.2598-3.0669 |
| Father with Hay Fever | | |  |  |  |  |  |  |  |
| 0 | No |  |  |  |  |  |  |  |  |
| 1 | Yes | 7 (43.8%) | 9 (56.2%) | 0.0235 (0.151) | 3.1795 | 1.1229-8.6455 | 0.0078 (0.53) | 6.1483 | 1.5706-23.803 |
| Father with wheeze | | |  |  |  |  |  |  |  |
| 0 | No |  |  |  |  |  |  |  |  |
| 1 | Yes | 9 (37.5%) | 15 (62.5%) | 0.0362 (0.178) | 2.46 | 1.0187-5.6189 | 0.1452 (0.759) | 2.6966 | 0.7014-10.3929 |
| Mother with asthma | | |  |  |  |  |  |  |  |
| 0 | No |  |  |  |  |  |  |  |  |
| 1 | Yes | 9 (20.5%) | 35 (79.5%) | 0.9471 (0.976) | 1.0257 | 0.4561-2.0831 | 0.5188 (0.91) | 0.7205 | 0.2484-1.8584 |
| Mother with hay fever | | |  |  |  |  |  |  |  |
| 0 | No |  |  |  |  |  |  |  |  |
| 1 | Yes | 4 (25%) | 12 (75%) | 0.6202 (0.806) | 1.3351 | 0.3699-3.8824 | 0.9933 (0.993) | 0.9928 | 0.1531-4.6821 |
| Mother with wheeze | | |  |  |  |  |  |  |  |
| 0 | No |  |  |  |  |  |  |  |  |
| 1 | Yes | 7 (25%) | 21 (75%) | 0.5093 (0.771) | 1.3407 | 0.5213-3.0577 | 0.6241 (0.91) | 1.3536 | 0.3755-4.3531 |
| **ENVIRONMENTAL FACTORS** | | | | | | | | | |
| Application of dung to farm fields | | |  |  |  |  |  |  |  |
| 0 | No |  |  |  |  |  |  |  |  |
| 1 | Yes | 5 (29.4%) | 12 (70.6%) | 0.3366 (0.694) | 1.678 | 0.5285-4.5855 | 0.7957 (0.91) | 1.1822 | 0.308-4.0361 |
| Cigarette smokers in the house | | |  |  |  |  |  |  |  |
| 0 | No |  |  |  |  |  |  |  |  |
| 1 | Yes | 16 (27.6%) | 42 (72.4%) | 0.1426 (0.441) | 1.5656 | 0.8371-2.7973 | 0.28 (0.907) | 1.4521 | 0.7183-2.8022 |
| Cooking area | |  |  |  |  |  |  |  |  |
| 0 | Outside living area | |  |  |  |  |  |  |  |
| 1 | Inside living area | 47 (13%) | 314 (87%) | 0 (0.002) | 0.4627 | 0.3201-0.6589 | 0.2035 (0.773) | 0.7141 | 0.423-1.1967 |
| Have a cat | |  |  |  |  |  |  |  |  |
| 0 | No |  |  |  |  |  |  |  |  |
| 1 | Yes | 61 (21.2%) | 227 (78.8%) | 0.5701 (0.806) | 1.1043 | 0.7806-1.5496 | 0.8441 (0.911) | 0.9584 | 0.6236-1.4582 |
| Have a cow | |  |  |  |  |  |  |  |  |
| 0 | No |  |  |  |  |  |  |  |  |
| 1 | Yes | 38 (18.7%) | 165 (81.3%) | 0.5893 (0.806) | 0.8968 | 0.5975-1.3201 | 0.2131 (0.773) | 0.7054 | 0.4016-1.208 |
| Have a dog | |  |  |  |  |  |  |  |  |
| 0 | No |  |  |  |  |  |  |  |  |
| 1 | Yes | 81 (20.9%) | 306 (79.1%) | 0.5793 (0.806) | 1.0956 | 0.792-1.5112 | 0.3558 (0.91) | 1.2095 | 0.8058-1.8086 |
| Have a hen | |  |  |  |  |  |  |  |  |
| 0 | No |  |  |  |  |  |  |  |  |
| 1 | Yes | 48 (22%) | 170 (78%) | 0.4114 (0.723) | 1.1675 | 0.8012-1.6796 | 0.8415 (0.911) | 0.9545 | 0.5998-1.4971 |
| Have a horse | |  |  |  |  |  |  |  |  |
| 0 | No |  |  |  |  |  |  |  |  |
| 1 | Yes | 21 (21.9%) | 75 (78.1%) | 0.6402 (0.806) | 1.13 | 0.6622-1.8548 | 0.72 (0.91) | 0.8855 | 0.4463-1.6957 |
| Have a pig | |  |  |  |  |  |  |  |  |
| 0 | No |  |  |  |  |  |  |  |  |
| 1 | Yes | 1 (20%) | 4 (80%) | 0.9972 (0.997) | 0.996 | 0.0508-6.781 | 0.6791 (0.91) | 0.5195 | 0.0114-7.3698 |
| Have a sheep | |  |  |  |  |  |  |  |  |
| 0 | No |  |  |  |  |  |  |  |  |
| 1 | Yes | 35 (22.2%) | 123 (77.8%) | 0.4728 (0.768) | 1.1641 | 0.7602-1.7464 | 0.461 (0.91) | 1.2265 | 0.7055-2.0949 |
| Source of water | |  |  |  |  |  |  |  |  |
| 0 | Piped |  |  |  |  |  |  |  |  |
| 1 | Well | 22 (27.8%) | 57 (72.2%) | 0.0703 (0.299) | 1.6177 | 0.9436-2.6875 | 0.4157 (0.91) | 1.2959 | 0.6851-2.3982 |
| 2 | River/Stream | 5 (22.7%) | 17 (77.3%) | 0.6852 (0.817) | 1.2328 | 0.4005-3.1687 | 0.8125 (0.91) | 1.1589 | 0.3095-3.6942 |
| Type of toilet | |  |  |  |  |  |  |  |  |
| 0 | Any toilet |  |  |  |  |  |  |  |  |
| 1 | None/Bush/Field | 16 (21.9%) | 57 (78.1%) | 0.6806 (0.817) | 1.1293 | 0.6141-1.9697 | 0.2062 (0.773) | 0.628 | 0.2967-1.2639 |
| Waste disposal | |  |  |  |  |  |  |  |  |
| 0 | Garbage Bin |  |  |  |  |  |  |  |  |
| 1 | Open Field | 53 (27.3%) | 141 (72.7%) | 0.0075 (0.073) | 1.6729 | 1.1422-2.4306 | 0.0391 (0.532) | 1.6149 | 1.0211-2.5424 |
| 2 | Pit | 25 (17.5%) | 118 (82.5%) | 0.8095 (0.89) | 0.9429 | 0.5744-1.4999 | 0.5908 (0.91) | 0.8613 | 0.4911-1.4639 |
| **HEMATOLOGICAL FACTORS** | | | | | | | | | |
| Hematocrit | |  |  |  |  |  |  |  |  |
| Continuous | | - | - | 0.0675 (0.299) | 1.0268 | 1.0036-1.0577 | 0.0303 (0.53) | 1.0594 | 1.0135-1.1295 |
| Hemoglobin | |  |  |  |  |  |  |  |  |
| Continuous | | - | - | 0.5104 (0.771) | 1.0329 | 0.9388-1.1389 | 0.7809 (0.91) | 0.9681 | 0.7767-1.2375 |
| Lymphocytes’ count | | |  |  |  |  |  |  |  |
| 0 | Normal |  |  |  |  |  |  |  |  |
| 1 | Low | 67 (18.1%) | 303 (81.9%) | 0.2423 (0.549) | 0.82 | 0.5858-1.1404 | 0.5128 (0.91) | 1.1381 | 0.7715-1.6767 |
| 2 | High | 2 (28.6%) | 5 (71.4%) | 0.64 (0.806) | 1.4833 | 0.2106-6.9753 | 0.7591 (0.91) | 1.3277 | 0.1682-7.5111 |
| Mean Corpuscular Hemoglobin | | |  |  |  |  |  |  |  |
| Continuous | | - | - | 0.0892 (0.337) | 0.9341 | 0.8635-1.0111 | 0.1072 (0.607) | 0.8717 | 0.729-1.0235 |
| Mean Corpuscular Hemoglobin Concentration | | | |  |  |  |  |  |  |
| Continuous | | - | - | 0.6017 (0.806) | 1.0184 | 0.9475-1.0947 | 0.5005 (0.91) | 1.0255 | 0.9495-1.1131 |
| Mean Corpuscular Volume | | |  |  |  |  |  |  |  |
| Continuous | | - | - | 0.4144 (0.723) | 0.9901 | 0.9669-1.0152 | 0.3974 (0.91) | 1.0271 | 0.9779-1.102 |
| Platelet count | |  |  |  |  |  |  |  |  |
| Continuous | | - | - | 0.3484 (0.697) | 1.0009 | 0.999-1.0027 | 0.5661 (0.91) | 1.0006 | 0.9984-1.0028 |
| Red Blood Cell count | | |  |  |  |  |  |  |  |
| Continuous | | - | - | 0.2192 (0.514) | 1.2035 | 0.8979-1.6221 | 0.2225 (0.773) | 0.6335 | 0.2912-1.3101 |
| White Blood Cell count | | |  |  |  |  |  |  |  |
| Continuous | | - | - | 0.0806 (0.322) | 0.943 | 0.8815-1.0056 | 0.4645 (0.91) | 0.9718 | 0.8987-1.0476 |
